# Kinetic models towards an enhanced understanding of diverse ADC conjugation reactions

**DOI:** 10.3389/fbioe.2024.1403644

**Published:** 2024-07-11

**Authors:** Jan Tobias Weggen, Ryan Bean, Kimberly Hui, Michaela Wendeler, Jürgen Hubbuch

**Affiliations:** ^1^ Institute of Process Engineering in Life Sciences, Section IV: Biomolecular Separation Engineering, Karlsruhe Institute of Technology (KIT), Karlsruhe, Germany; ^2^ Purification Process Sciences, BioPharmaceuticals Development, R&D, AstraZeneca, Gaithersburg, MD, United States

**Keywords:** antibody-drug conjugate (ADC), conjugation reaction, kinetic model, site-specific conjugation, interchain disulfide, cysteine-conjugates, payload

## Abstract

The conjugation reaction is the central step in the manufacturing process of antibody-drug conjugates (ADCs). This reaction generates a heterogeneous and complex mixture of differently conjugated sub-species depending on the chosen conjugation chemistry. The parametrization of the conjugation reaction through mechanistic kinetic models offers a chance to enhance valuable reaction knowledge and ensure process robustness. This study introduces a versatile modeling framework for the conjugation reaction of cysteine-conjugated ADC modalities—site-specific and interchain disulfide conjugation. Various conjugation kinetics involving different maleimide-functionalized payloads were performed, while controlled gradual payload feeding was employed to decelerate the conjugation, facilitating a more detailed investigation of the reaction mechanism. The kinetic data were analyzed with a reducing reversed phase (RP) chromatography method, that can readily be implemented for the accurate characterization of ADCs with diverse drug-to-antibody ratios, providing the conjugation trajectories of the single chains of the monoclonal antibody (mAb). Possible kinetic models for the conjugation mechanism were then developed and selected based on multiple criteria. When calibrating the established model to kinetics involving different payloads, conjugation rates were determined to be payload-specific. Further conclusions regarding the kinetic comparability across the two modalities could also be derived. One calibrated model was used for an exemplary *in silico* screening of the initial concentrations offering valuable insights for profound understanding of the conjugation process in ADC development.

## 1 Introduction

Targeted anticancer therapeutics are becoming increasingly prevalent in the field of biopharmaceuticals. One important class in this toolbox are antibody-drug conjugates (ADCs), which consist of a conventional monoclonal antibody (mAb) chemically coupled with a highly potent small-molecule (so called “drug” or “payload”). The success of an ADC depends on conjugating a specific number, typically between two to eight, of cytotoxic payload onto a mAb, determining the final ADC potency and toxicity (Chau et al., 2019). Related to this, one facet is the choice of the conjugation strategy which controls critical quality attributes (CQA) of the final product, such as the drug-to-antibody ratio (DAR) and drug load distribution (DLD) (Ponziani et al., 2020; Dean et al., 2021). To overcome problems associated with unfavorable DAR heterogeneities and to improve the overall potency, site-specific conjugation or more hydrophilic linkers have been developed (Panowski et al., 2014; Joubert et al., 2020). On the downside, these continuous advances hinder the development of a standardized platform process, and thus increase the time and effort to develop a scalable and robust manufacturing process (Hutchinson et al., 2018; Matsuda and Mendelsohn, 2021).

Quality by Design (QbD) is increasingly expected by regulatory agencies, aiming to ensure consistent product quality and improve process understanding (Anurag and Winkle, 2009). One major aspect in QbD is the utilization of modeling techniques which has been proven to be an invaluable tool for gaining insights into complex systems and optimizing manufacturing operations (Roush et al., 2020; Babi et al., 2022). Process modeling enables parametrization of (bio)chemical effects dominating bioprocesses and hence, understanding the impact of such effects *in silico*. In essence, process models serve as a basis for digital and automation technologies that can accelerate the development of efficient and robust manufacturing processes (Narayanan et al., 2020).

For biochemical reactions, one particularly important aspect of process modeling is kinetic modeling, which focuses on characterizing the rates and complex mechanisms of reactions involved. In contrast to statistical approaches, such as Design of Experiment (DoE) which purely rely on the statistical relationship between input and output variables, kinetic models provide a quantitative description of the underlying reaction kinetics, elucidating the impact of various factors such as temperature, pH, reactant concentrations (Taylor et al., 2022). Previous work has demonstrated the successful establishment of kinetic models in the area of bioprocessing, e.g., for biomass conversion (Vollmer et al., 2022), fermentation (Kyriakopoulos et al., 2018) or small-molecule synthesis (Ashworth et al., 2019), and with a special focus on protein modification, e.g., for protein PEGylation (Pfister et al., 2016), antibody oxidation (Tang et al., 2020) or antibody reduction for ADCs (Nayak and Richter, 2023). The challenge of creating a kinetic model usually centers around finding the correct rate laws for each individual reaction step and their corresponding rate constants. This can be cumbersome due to the presence of multiple interacting species or complex reaction networks. To find the most reliable process model among possible model candidates, different techniques have been proposed in the literature to assess the quality of the estimated parameters (e.g., parameter identifiability analysis or Fisher Information Matrix) and quantifying the output uncertainty (Sin et al., 2009; Anane et al., 2019; Rodman and Gerogiorgis, 2020).

In ADC manufacturing, the conjugation reaction represents a key step as it generates the ADC molecule (Abdollahpour-Alitappeh et al., 2019). Comprehensive understanding of the kinetics of this reaction step is vital for process developers, as it enables the minimization of payload usage, thereby reducing the cost-of-goods. Consequently, employing less payload also facilitates the removal of free unconjugated payload, which is crucial to minimize toxicity of the final product (Fernandez-Cerezo et al., 2023). The currently available literature guiding process development for ADCs is scarce (Matsuda et al., 2020). Typically, DoE methodologies are utilized to gain knowledge about reaction parameters, such as reactant concentrations, temperature, time or pH, affecting the conjugation process (Stump and Steinmann, 2013), which can be augmented when high-throughput screenings (Andris et al., 2018) or continuous flow reactors (Nakahara et al., 2022) are used to automatize experimental work. However, DoEs cannot provide a deep understanding of the molecular or chemical mechanisms that drive the reaction. Andris et al. (2019) developed a mechanistic kinetic model for a site-specific conjugation reaction using a pseudo payload. The study showcases the benefit of the kinetic model as a versatile *in silico* decision tool for the investigation and development of the conjugation reaction. In subsequent studies, we could show that conjugation kinetic models realize their full potential by coupling a kinetic model with computational fluid dynamics (CFD) to study large-scale conjugation reactions (Weggen et al., 2023), and enable real-time monitoring of non-observable ADC species during the reaction through combination with UV/Vis spectroscopy via an extended Kalman Filter (Schiemer et al., 2023). However, applications of this kinetic model are still limited due to various reasons: 1) The reliance on an analytical technique that is performed under native (non-denaturing) conditions which is usually limited to ADCs with low DAR values; 2) a relatively small concentration range up to 2.5 g/L, whereas actual operational conditions may be higher, typically around 20 g/L; 3) the unproven transferability of the kinetic model to other payloads; and 4) the unverified application to alternative conjugation modalities such as interchain cysteine conjugation.

This study focuses on mechanistic modeling of the conjugation kinetic for two prominent cysteine-based modalities, site-specific (DAR 2) and interchain disulfide (DAR 8) conjugation, which represent a main part of current conjugation chemistries. Both modalities rely on the conjugation to reactive cysteines, but differ in the number and location of the reactive cysteines on the mAb. Batch and fed-batch conjugation kinetics are generated across a broad range of initial mAb concentrations and drug excess. Time-course samples are analyzed using a widely employed and easily implemented reducing reversed phase ultrahigh performance liquid chromatography (RP-UHPLC) method that separates the conjugated mAb into its respective subunits prior to analysis, thereby producing well-resolved chromatograms that illustrate conjugation state of heavy and light chains. Consequently, the kinetic data represents the conjugation trajectories of heavy and light chains. Due to the different quantities of reacting species in the two modalities, multiple kinetic model candidates are proposed and the optimal number of kinetic rates for each model type is chosen based on multiple criteria: parameter identifiability, parameter uncertainty and prediction errors. In addition, absorbance measurements are conducted to determine the stability of each payload in conjugation buffer. Ultimately, an *in silico* screening is performed accounting for the effects of reactant concentration and exemplifying the usage for process optimization.

## 2 Materials and methods

### 2.1 Experimental conjugation kinetic studies

The kinetic datasets encompass two distinct ADC modalities. The datasets 1 and 2 use an engineered IgG1 mAb—ADC1—with two inserted cysteines in the hinge region for a site-specific “DAR 2” conjugation to engineered cysteines. The datasets 3 and 4 use two different IgG1 mAbs—ADC2 and ADC3—for a “DAR 8” conjugation to reduced cysteines that are usually engaged in interchain disulfide bonds. Table 1 summarizes molecules and conjugation conditions. Datasets 1, 2 and 4 were generated at *AstraZeneca* and dataset 3 at *Karlsruhe Institute of Technology (KIT)*. Minor differences due to different raw materials in chemicals and analytical devices are expected.

**TABLE 1:** Summary of experimental conditions of all conjugation runs using different ADC modalities and payloads. A detailed overview for the individual experiments is given in the Supplementary Material.

Dataset	ADC	Type	Payload	$c_{mAb}\;/\;\left(g\;L^{-1}\right)$	Molar drug excess	Drug addition	No. of conditions
1	ADC1	DAR 2	Drug1	1.5–10	1x–8x	Batch	8
2	ADC1	DAR 2	NPM	1.5–3	3x–5x	Batch	4
3	ADC2	DAR 8	NPM	1.5–3	6x–13x	Batch/Fed-Batch	10
4	ADC3	DAR 8	Drug2	1.5 & 20	11x & 14x	Batch	4

#### 2.1.1 Chemicals, ADCs and functionalization steps

For DAR 2 conjugation of ADC1, the antibody was initially fully reduced through treatment with tris (2-carboxyethyl) phosphine hydrochloride (TCEP, EMD Millipore), followed by a buffer exchange using Vivaspin 20 (30 kDa MWCO, Cytiva) and a re-oxidation of the interchain disulfides with (L)-dehydroascorbic acid (DHAA, Sigma-Aldrich). Conjugation was performed using a maleimide-functionalized payload. Two payloads were compared for ADC1, a cytotoxic payload (“Drug1”) and a nontoxic surrogate N-(1-pyrenyl)-maleimide (NPM, Merck KGaA). For DAR 8 conjugation of ADC2 and ADC3, a full reduction of the interchain disulfides with TCEP was performed. Conjugation for ADC2 was carried out with NPM, while for ADC3 another cytotoxic payload (“Drug2”) was used. For reaction quenching, N-acetyl cysteine (NAC, Merck KGaA) was used. All payloads were dissolved in DMSO (Sigma-Aldrich). All other solutions were prepared with 20 mM sodium phosphate buffer (J.T. Baker), 1 mM EDTA (EMD Millipore), pH 7.0. For sample pretreatment for DAR analysis, samples were diluted with denaturing buffer containing guanidine HCl (Thermo Fisher), Tris (Thermo Fisher), EDTA (EMD Millipore) at pH 7.6 and reduced with dithiothreitol (DTT, Thermo Fisher). For the sample analysis, a RP-UHPLC (Agilent Technologies) with acetonitrile (VWR) and HPLC water (VWR) with 0.1% (v/v) trifluoroacetic acid (Thermo Scientific) as mobile phases were used.

#### 2.1.2 Conjugation kinetics

For site-specific DAR 2 conjugation (ADC1), the antibody was initially treated with the reducing agent TCEP, which reduces both the engineered inserted cysteine residues, as well as cysteines engaged in interchain disulfide bonds. The reducing agent was subsequently removed and the buffer was exchanged, before the antibody was mildly re-oxidized with DHAA which allows re-formation of interchain disulfide bonds, leaving only the two inserted cysteines available for conjugation. DAR 2 conjugation were performed by adding either Drug1 or NPM solution to the re-oxidized mAb solution in a microcentrifuge tube at 1 mL scale. Selected conditions were performed in duplicates. For conjugation to reduced interchain disulfides, the native mAb was completely reduced. In the DAR 8 conjugations of ADC2 ten out of thirteen kinetics were performed in fed-batch mode, where drug solution was continuously added to resolve the fast time-course of the individual reacting species. These reactions were performed in centrifuge tubes at a liquid volume of 4 mL and the required volume of payload solution was constantly added with a syringe pump (Nemesys S, Cetoni GmbH) over a defined period of time (10, 20 or 30 min) to the stirred reaction solution. The remaining batch conjugations were performed at 1 mL scale. For ADC3, batch conjugation and another payload (Drug2) were used. An overview of the experimental conditions for the conjugation kinetics is given in Table 1. A detailed overview of all experimental conditions is given in Supplementary Table S1. To acquire conjugation kinetics, samples were taken at discrete time points over the course of 1 h, transferred to a microcentrifuge tube and immediately quenched by adding a 12x molar excess over payload of NAC solution to terminate the conjugation reaction.

#### 2.1.3 Sample treatment and reference analytics

To determine the DAR and the DLD of each sample a reducing RP method was used. A sample treatment was conducted to reduce the conjugated ADC molecule into heavy and light chains. Sample concentrations were adjusted to 1 mg/mL using ultrapure water. Samples were then mixed with denaturing buffer and DTT solution before incubating over 30 min at 37°C on an orbital shaker (650 rpm). For the RP-UHPLC analysis, 10 µL of sample were injected onto a BioResolve RP mAb polyphenyl column (2.1*150 mm, 2.7 µm, Waters Corporation). An identical method, including flow rate, gradient combination and buffer compositions, was used at AZ and KIT, as described previously (Cao et al., 2019). The peak areas in the resulting chromatograms were manually determined. The molar concentrations of the corresponding conjugated and unconjugated heavy or light chains were calculated by normalizing the peak area of light/heavy chain to the respective concentration in the reaction.

#### 2.1.4 Payload depletion study

As the inactivation of the used surrogate NPM was reported in a previous study (Andris et al., 2019), a depletion study for each of the tested payloads was conducted in order to determine the individual stability in conjugation buffer. For that purpose, DMSO-dissolved payload solution was added to conjugation buffer reaching a payload concentration of 0.1 mM and 10% DMSO in a 1.5 mL microcentrifuge tube. The solution was allowed to mix on a tube rotator. Samples were taken over the course of 1 h and the absorbance was measured using the UV/Vis function on the Stunner light scattering instrument (Unchained Labs). For NPM, the course of the absorbance was directly measured in a cuvette using a spectrophotometer (Spectrostar Nano, BMG Labtech). To verify the model assumption that depleted payload becomes unreactive for conjugation, the payloads NPM and Drug1 were separately dissolved in conjugation buffer (10% DMSO, 0.1 mM) and mixed for 1 h on a tube rotator instead of using it immediately for conjugation.

### 2.2 Conjugation kinetic model development

#### 2.2.1 Modeling reduced conjugation kinetics and reaction schemes

**FIGURE 1:**
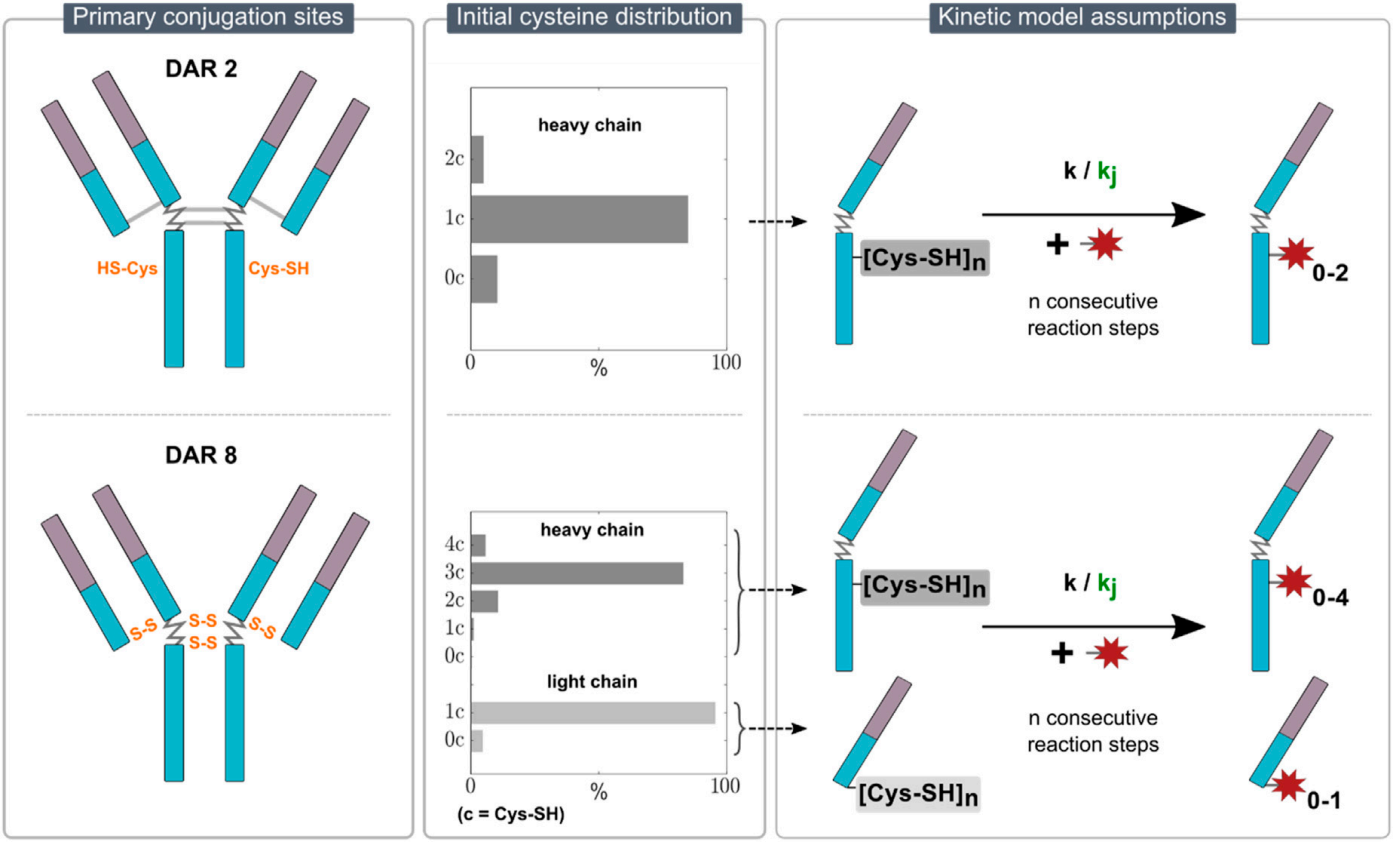
Overview of the kinetic model assumptions for DAR 2 (top row) and DAR 8 (bottom row) conjugation. The left panel illustrates the primary conjugation sites for each ADC in orange. The middle panel indicates the initial cysteine distribution for each ADC. The right panel presents the proposed stepwise conjugation reaction schemes of the payload to n reactive thiols on either light/heavy chain with either one kinetic rate (simple model) or j kinetic rates (detailed model) for each ADC.

Due to the sample treatment, which reduces the mAb in each sample and consequently eliminates information about the intact ADC species, the kinetic models employed in this study describe the conjugation reaction to the individual mAb chains. For the DAR 2 mAb, only conjugation to heavy chain was observed. This is expected, as the inserted cysteine residues are located in the hinge region of the heavy chain. In contrast, for DAR 8, conjugation to both heavy and light chain was observed as expected for stochastic conjugation. Given the absence of analytical data to resolve positional isomers, iso-reactivity for different binding locations on the heavy chain was assumed. These constraints narrow the possible rate equations down to consecutive reactions schemes describing the heavy chain as a “black box” with a certain number of available binding sites and exclude parallel schemes. The primary conjugation sites for the two ADC types with an overview of the assumed kinetic models are illustrated in Figure 1.

##### 2.2.1.1 Initial cysteine distribution

To align with the observed DLD at the end of the reaction, i.e., the ratio of differently conjugated heavy and light chains, an ADC-specific initial reactive cysteine distribution of the starting mAb material was set prior parameter estimation. With regards to the DAR 2 conjugation, the main product in the final DLD is H1 (heavy chain +1 drug). To model the amounts of unconjugated and over-conjugated heavy chains, H0 and H2, respectively, we assumed an initial cysteine distribution of heavy chains with zero, one and two reactive cysteines, namely, ${H0}_{0c}$ , ${H0}_{1c}$ , and ${H0}_{2c}$ . Over-conjugation in DAR 2 ADCs is thought to occur on cysteines as discussed in Cao et al. (2019). On the contrary, for DAR 8, this distribution contains heavy chains with up to four and light chains with up to one reactive cysteine, while ${H0}_{3c}$ (heavy +3 drug) and ${L0}_{1c}$ (light chain +1 drug) are the main species. In this case the distribution is primarily controlled by the conditions in the reduction reaction, which were kept constant in this study. It is important to note, that the presence of heavy chain with four drugs in the case of DAR 8 depicts unspecific over-conjugation that is likely to be attributed to additional “mis-alkylation” of the payload to other residues such as lysine, which was demonstrated to occur for other model proteins (Paulech et al., 2013). This mis-alkylation appears to be marginal in the range of the studied drug excess, which led to the assumption that it only occurs in addition to the primary conjugation of the three cysteines. The chemical group of the fourth binding site is unknown, but is declared as being part of the initial cysteine distribution for the sake of simplicity. Furthermore, as the distribution might slightly vary for each mAb-drug combination, it was determined specifically for each ADC. Consequently, the initial cysteine distribution was determined experimentally per ADC based on the average distribution (%H0, %H1, … ) at the final steady-state of the reaction ( $t_{f}=60\;\min$ ) in experiments with sufficient drug excess to reach full conjugation of all available cysteines, i.e., drug excess > 3x for DAR 2 and drug excess > 8x for DAR 8. As an example, the percentage of $H0_{1c}$ was calculated from the averaged percentage of $H1_{t_{f}}$ among all heavy chains. The determined distribution was then used to calculate the molar concentration of the various initial species within the starting mAb. Example distributions for DAR 2 and DAR 8 are shown in Figure 1.

##### 2.2.1.2 Rate equations

For the conjugation reaction in a perfectly mixed system with continuous drug addition over a fixed period, the dilution rate due to added drug solution has to be considered. Therefore, the rate equation for a reaction species *i* can be expressed as:

$$\frac{dc_{i}}{dt}=r_{conj,i}-\frac{c_{i}\left(t\right)}{V_{l}\left(t\right)}q_{in}\;,$$ (1)

where $c_{i}$ represents the molar concentration of the *i*th species, $r_{\text{conj},i}$ is the conjugation reaction rate of the *i*th species, $V_{l}$ represents the reaction volume and $q_{\text{in}}$ is the feed flow rate. In case of the free drug, the second term in Eq. 1 is changed to $+\frac{c_{\text{drug},\text{in}}}{V_{l}\left(t\right)}q_{\text{in}}$ , where $c_{\text{drug},\text{in}}$ is the drug concentration in the feed. The change in volume can be described with Eq. 2:

$$\frac{dV_{l}}{dt}=q_{in}\;.$$ (2)

To account for the sampling at discrete time points, the equation is integrated until each sampling time point and the volume is subtracted by the sample volume. In case of a batch conjugation reaction, Eq. 1 simplifies to Eq. 3:

$$\frac{dc_{i}}{dt}=r_{conj,i}\;.$$ (3)

To describe the reaction rates, some assumptions were made: conjugation refers to the second-order reaction of the maleimide of a functionalized payload with the sulfhydryl residue (SH-group) of a reactive cysteine. The reaction was assumed to be irreversible as no de-conjugation was observed and temperature effects on the conjugation kinetic were neglected as the temperature did not show noticeable effects on the kinetics. Moreover, a payload-specific depletion rate $k_{\text{drug}}$ caused by, for example, unspecific adsorption of the hydrophobic molecule to vessels wall, was considered. Based on these assumptions, a system of ordinary differential equations (ODE) describing the rate equations was formulated. Two model candidates, for both DAR 2 and DAR 8 conjugation reaction, varying in their degree of complexity were proposed: Either “simple” conjugation rates assuming one kinetic rate for all conjugation steps, or “detailed” conjugation rates assuming individual kinetic rates for each sequential reaction step. The basic reaction schemes for both DAR modalities can be found in the Supplementary Material. As an example, the seven ODEs for the detailed model for the DAR 2 reaction are given in Eqs 4–10:

$$\frac{dc_{H0_{2c}}}{dt}=-k_{1}c_{H0_{2c}}c_{drug}$$ (4)

$$\frac{dc_{H0_{1c}}}{dt}=-k_{1}c_{H0_{1c}}c_{drug}$$ (5)

$$\frac{dc_{H0_{0c}}}{dt}=0$$ (6)

$$\frac{dc_{H1_{2c}}}{dt}=-k_{2}c_{H1_{2c}}c_{drug}+k_{1}c_{H0_{2c}}c_{drug}$$ (7)

$$\frac{dc_{H1_{1c}}}{dt}=k_{1}c_{H0_{1c}}c_{drug}$$ (8)

$$\frac{dc_{H2_{2c}}}{dt}=k_{2}c_{H1_{2c}}c_{drug}$$ (9)

$$\frac{dc_{\text{drug}}}{dt}=-k_{1}c_{H0_{2c}}c_{\text{drug}}-k_{1}c_{H0_{1c}}c_{\text{drug}}-k_{2}c_{H1_{2c}}c_{\text{drug}}-k_{\text{drug}}c_{\text{drug}}\;.$$ (10)

Here, $c_{H}$ denotes the molar concentration of the heavy chain with the number indicating the number of conjugated drugs and the index indicates the number of initial available cysteines. Furthermore, $c_{\text{drug}}$ represents the concentration of the payload, $k_{j}$ the reaction rate for the *j*th conjugation step and $k_{\text{drug}}$ the depletion rate of the specific payload. The system of ODEs for the DAR 8 kinetic models can be found in the Supplementary Material.

##### 2.2.1.3 Payload depletion rate

Initially, the estimation of the model parameter $k_{\text{drug}}$ led to large confidence intervals and correlation coefficients to the other conjugation rates. This could be attributed to the absence of direct kinetic data for $c_{\text{drug}}$ in combination with the interconnected system of ODEs with regards to $c_{\text{drug}}$ being present in all ODEs (see Eqs 4–10). Consequently, the depletion rate was considered non-identifiable given the available kinetic data and was separately determined by UV/Vis spectroscopy (cf. Chapter 2.1.4). The UV/Vis-determined depletion rate for each payload was then set constant for the subsequent estimation of the remaining conjugation rates.

#### 2.2.2 Data handling

All runs for one mAb and one payload were summarized as a kinetic dataset resulting in four distinct datasets as outlined in Table 1. All replicate kinetics were averaged. The dataset involving Drug1 was used as the basis for DAR 2 model candidate selection. A manual split in six training and two test runs was done. The dataset with NPM was used for external model re-calibration to compare the conjugation kinetic rates of the payloads.

In the case of DAR 8, two individual datasets were available: The first one comprised fed-batch and batch kinetics of ADC2 with the surrogate drug NPM and was utilized for DAR 8 model candidate selection. Similarly, a manual split in ten training and three test runs was performed. The second external dataset, containing batch kinetics of ADC3 and Drug2, was later used for the *in silico* screening. A detailed overview of all runs and the train/test split is provided in the Supplementary Table S1.

As explained above, a sufficient drug excess, such as 3x for DAR 2 and 11x for DAR 8, resulted in a saturation of both the DAR and the DLD at the steady state of the conjugation reaction. Lowering the drug excess led to lower DAR value and changing DLD. The saturated DAR and DLD is assumed as being unique to every mAb and payload. With regards to the modeling assumptions, the average DLD of kinetics with sufficiently large drug excesses was considered to compute the initial cysteine distribution for each individual kinetic subset.

#### 2.2.3 Parameter estimation

All simulations were performed in Matlab R2023a (The MathWorks Inc.). Maximum likelihood estimation of the kinetic rates in each model was conducted by minimizing the squared error between model predicted and experimental concentrations using the in-built *lsqnonlin* function. To account evenly for the entire concentration range, each run was normalized using a scaling factor corresponding to the maximum concentration in the respective run. Model predictions were performed based on the initial concentrations of the starting mAb, the initial cysteine distribution and payload concentration. The ODE system is numerically solved using the *ode15s* solver.

#### 2.2.4 Model candidate selection criteria

The selection of the most appropriate kinetic model candidate was conducted based on various metrics, namely, the quality of the estimated parameters, parameter identifiability ranking and model errors regarding the cross-validation of the training data and the test set.

##### 2.2.4.1 Uncertainty of the estimated parameters

The uncertainty of the estimated parameters was evaluated based on their statistical uncertainty. First, the parameter covariance matrix $\text{cov}\left(\hat{\theta }\right)$ was calculated using the Jacobian matrix J assuming independent measurement errors with Gaussian white noise according to Eq. 11:

$$cov\left(\hat{\theta }\right)=s^{2}{\left(J^{\prime }∙J\right)}^{-1},$$ (11)

with Eq. 12

$$s^{2}=\frac{\sum_{i=1}^{N}{\hat{\epsilon }}^{2}}{n-p}\;,$$ (12)

where $s^{2}$ denotes the variance of the error, $\hat{\epsilon }$ is the error between predicted and measured concentrations, n indicates the number of samples and p is the number of parameters. The parameter standard deviation σ is calculated from the diagonal elements of the parameter covariance matrix given as Eq. 13:

$$\sigma =\sqrt{diag\left(cov\left(\hat{\theta }\right)\right)}\;.$$ (13)

Typically, parameter estimates can be considered reasonable, when the parameter standard deviations values is below 25% relative to the parameter estimate (Sin et al., 2016). The confidence intervals of the parameters were estimated using *nlparci* in Matlab.

##### 2.2.4.2 Parameter identifiability analysis

Local sensitivity analysis employing the one factor at a time (OAT) method was conducted in accordance to Sin et al. (2009). In this method, each model parameter, i.e., the kinetic rates, is systematically varied holding others constant, and the resulting impact on the model’s output is observed. In practice, sensitivities for each model parameter are calculated using the first-order derivative of the model output with respect to the parameter. For each parameter, the derivatives are determined numerically by perturbing the parameter of interest by 10% of its nominal value. The sensitivities are then averaged over time and relative sensitivities $sr_{j,i}$ with respect to each species *i* and each model parameter *j* are calculated by accounting for the parameter nominal value ${\hat{\theta }}_{j}$ according to Eq. 14:

$$sr_{j,i}=\frac{\partial y_{i}\left(t\right)}{\partial \theta_{j}}\theta_{0}\;.$$ (14)

By computing the square root of the mean of all sensitivities over all runs, the parameter significance values were calculated according to Eq. 15:

$$\delta^{msqr}=\sqrt{\frac{1}{N}\sum_{i=1}^{N}\left(sr_{j,i}\right)}\;.$$ (15)

Summing up all significance values returns the $\sum \delta^{msqr}$ value, allowing for a parameter importance ranking according to their impact on the model’s output. Thus, this approach aids in identifying which kinetic rates are identifiable based on their impact. The parameter identifiability analysis was performed for the two detailed conjugation models using the previously calibrated kinetic rates.

##### 2.2.4.3 Model error

The estimated kinetic rates were then used to predict the kinetic and the resulting model error regarding the species *i* was evaluated using the root mean squared error $\left(\text{RMS}E_{i}\right)$ given as Eq. 16:

$${RMSE}_{i}=\sqrt{\frac{1}{N}\sum_{i=1}^{N}{\left(c_{i}\left(t\right)-\tilde{c_{i}}\left(t\right)\right)}^{2}}\;,$$ (16)

where $c_{i}\left(t\right)$ denotes the predicted species concentration, ${\tilde{c}}_{i}\left(t\right)$ is the measured species concentration and N indicates the number samples. Afterwards, the $\text{RMS}E_{i}$ values of each species were averaged to one RMSE value for each subset. To assess the capability of the model candidates to generalize and extrapolate, both the prediction error based on cross-validation of the training data and on the independent test set were conducted. Cross-validation was performed based on *leave-one-run-out* scheme using the training runs. This leads to two error metrics RMSECV and RMSEP .

#### 2.2.5 In-silico screening for influence of initial mAb and payload concentrations

An *in silico* screening was conducted for the calibrated DAR 8 kinetic model (ADC3 + Drug2) in order to observe the model output at variable initial concentrations of mAb and payload. The model outputs were the final DAR and the concentration of unreacted payload after a reaction time of 30 min as well as the necessary reaction time to reach the final DAR below a threshold of 1%. A systematic screening with regards to the three model outputs was done by independently varying the initial mAb concentration between 1.5 and 10 g/L and the drug excess between 5 and 14 M excess while keeping the other constant.

## 3 Results

First, the UV/Vis measurements for the different payloads and the determination of the payload depletion rates are presented. Second, the bulk of this work demonstrates the selection of suitable conjugation kinetics models. Additionally, the established kinetic models are assessed regarding their ability to predict experimental kinetics for DAR 2 and 8 with high accuracy. An additional test study was conducted to evaluate the model’s capability for *in silico* screening using the DAR 8 model as example.

### 3.1 Payload depletion

**FIGURE 2:**
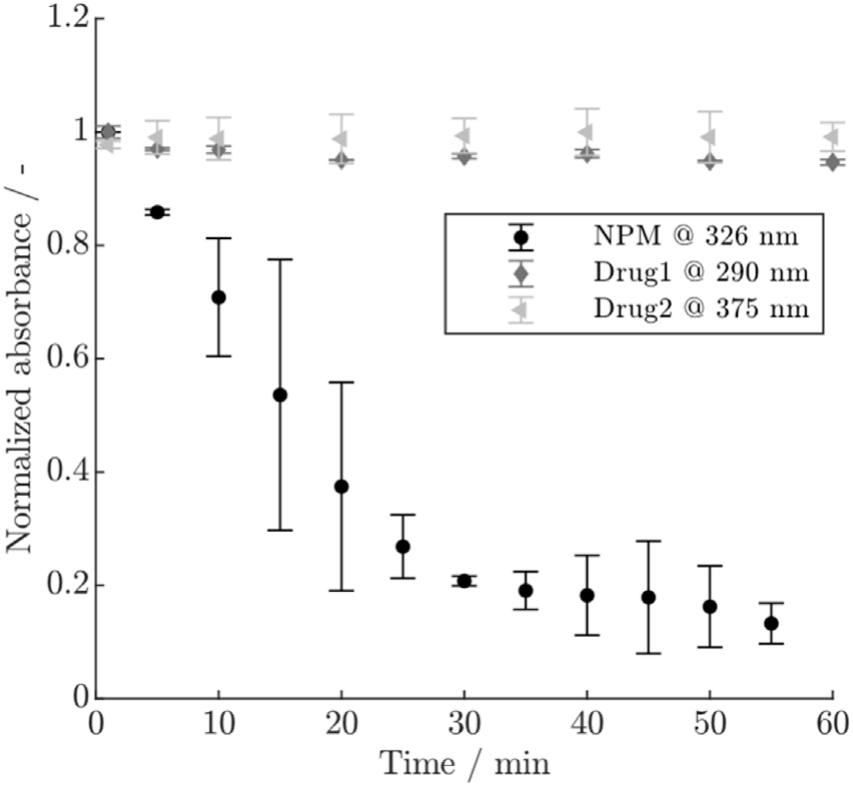
Normalized absorbance for the three payloads NPM, Drug1 and Drug2 at different wavelengths over time. The error bars indicate the standard deviation of the duplicate measurements.

The course of the normalized absorbance for the three payloads is shown in Figure 2. For NPM, a rapid decrease in absorbance is visible within 1 h, accompanied by larger error bars, whereas Drug1 exhibits only a minor decrease, and no remarkable change is visible for Drug2. The conjugation experiments using either NPM or Drug1 which was previously hold in conjugation buffer for 1 h, showed that for NPM the achieved DAR is strongly reduced as opposed to Drug1 which still reaches a typical DAR value in the range of 1.8–1.9 (see Supplementary Figure S1). Since the exact mechanism of the payload depletion is unknown, a first-order reaction was assumed to describe the depletion of NPM and Drug1, similar to Pfister et al. (2015). The depletion rate for NPM could be approximated by plotting the natural logarithm of the absorbance over time (cf. Supplementary Figure S2). Hereby, a depletion rate of $k_{\text{drug},\text{NPM}}=0.041\;s^{-1}$ could be derived using the slope of the linear regression curve. For Drug1 a depletion rate of $k_{\text{drug},\text{Drug}1}=0.001\;s^{-1}$ was assumed, as this value showed to agree within the modeling workflow. The depletion rate for Drug2 was assumed to be zero.

### 3.2 Model complexity evaluation

Two types of kinetic models with varying complexity, namely, simple and detailed, for either DAR 2 or DAR 8 conjugation kinetics were evaluated with regards to the parameter importance (assessed through the parameter identifiability analysis), parameter confidence and model error. The entire set of estimated parameters including their standard deviation and confidence intervals as well as model error are listed in Table 2.

**TABLE 2:** Results of the parameter estimation for the kinetic model candidates for either DAR 2 or DAR 8 conjugation. Parameter estimation include parameter estimate $\left(\hat{\theta }\right)$ , parameter standard deviation ( $\left.\sigma \right)$ and lower (lb) and upper bound (ub) of the confidence intervals (all in $L\;{\left(mmol\;s\right)}^{-1}$ units). Model error include cross-validation (RMSECV) and test errors (RMSEP) in $µmol\;L^{1}$ .

ADC	Model	Parameter estimation					Model error	
		Parameter	${\hat{\theta }}_{j}$	$\sigma_{j}$	lb	ub	RMSECV	RMSEP
DAR 2	Simple (1k)	$k_{1}$	0.251	0.023	0.204	0.298	1.863	3.188
	Detailed (2k)	$k_{1}$	0.297	0.027	0.242	0.351	1.808	3.207
		$k_{2}$	1.712	6.585	−11.515	14.939		
DAR 8	Simple (1k)	$k_{1}$	1.733	0.018	1.697	1.768	1.213	1.339
	Detailed (4k)	$k_{1}$	1.220	0.003	1.214	1.227	0.620	0.964
		$k_{2}$	1.853	0.006	1.841	1.865		
		$k_{3}$	5.117	1.015	3.101	7.133		
		$k_{4/5}$	2.312	0.356	1.605	3.020		

**FIGURE 3:**
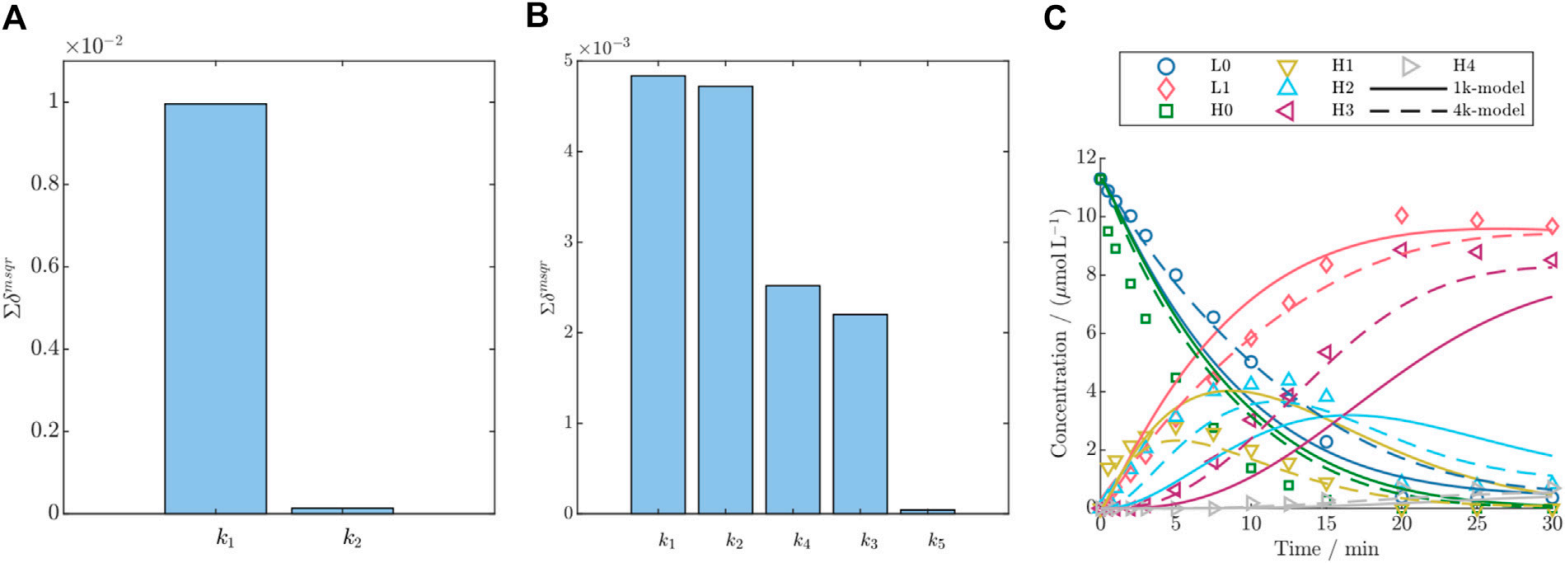
Parameter importance ranking according to the summed significance ${\sum \delta }^{msqr}$ for the detailed DAR 2 model **(A)** and DAR 8 **(B)**, and **(C)** comparison of the predictions for the simple (1k) and detailed (4k) DAR 8 kinetic model vs the experimental data for an example training run with 1.5 g/L ADC2 + 11x NPM and $t_{f}$ = 30 min.

For DAR 2, the difference between the two models is the addition of a second rate to account for an independent second conjugation step. The rate $k_{1}$ displays small standard deviation and narrow confidence intervals for both model candidates as opposed to $k_{2}$ which exhibits large confidence intervals. At the same time, the addition of a second rate did not improve the average prediction performance considerably as can be seen from similar RMSECV and RMSEP values between the two parameter subsets. The parameter importance ranking for the detailed DAR 2 model demonstrated that the rate $k_{2}$ has almost zero effect on the model output as opposed to the rate $k_{1}$ which has remarkably higher importance as shown in Figure 3A. Hence for the further modeling purpose the simple (1k) model was chosen for the modeling of DAR 2 conjugation kinetic.

With respect to the DAR 8 model, initially a model with five conjugation rates accounting for each conjugation step were used. The parameter importance ranking demonstrated that the last conjugation rate $k_{5}$ that accounts exclusively for the over-conjugation step is non-identifiable compared to the other rates, as shown in Figure 3B. Consequently, this rate was combined with the rate for the previous conjugation step $k_{4}$ (referred as $\left.k_{4/5}\right)$ yielding a lumped version of the model having four kinetic rates. The comparison for the simple and detailed model regarding the parameters and model error is given in Table 2. For both DAR 8 models, the confidence intervals are in acceptable ranges, while the intervals relative to the mean for $k_{1}$ and $k_{2}$ are with values below 1% considerably smaller than for $k_{3}$ and $k_{4}$ with values around 25%. It could be shown that the detailed model reduces the RMSECV and RMSEP by approximately 49% and 37%, respectively. To further examine the effect of the two models on the prediction performance, a comparison of the two model’s predictions is shown versus the experimental data for one example run in Figure 3C. The experimental data indicate a more rapid decrease of H0 compared to L0, as well as a sequential formation of the species H1, H2, H3, and H4 in the mentioned order. It can be clearly seen that the detailed (4k) model aligns with the kinetics of all single species more precisely compared to the simple (1k) model. Specifically, the dynamic behavior of H1, H2, and H3 is remarkably better captured by the detailed model. As shown in Table 2, the resulting estimated rate for conjugation to light chain ( $k_{1}$ ) is slower than the rates representing conjugation to heavy chain ( $k_{2}$ – $k_{4}$ ). For the stepwise conjugation to heavy chain, the second step ( $\left.k_{3}\right)$ seems to be faster than the first step while the third/fourth step ( $k_{4}$ ) is again slower. The enhanced performance of the detailed model was observed throughout all runs. Therefore, the detailed (4k) model was used for the subsequent modeling of the DAR 8 conjugation reactions.

### 3.3 Modeling site-specific DAR 2 conjugation kinetics

#### 3.3.1 Batch conjugation kinetic

**FIGURE 4:**
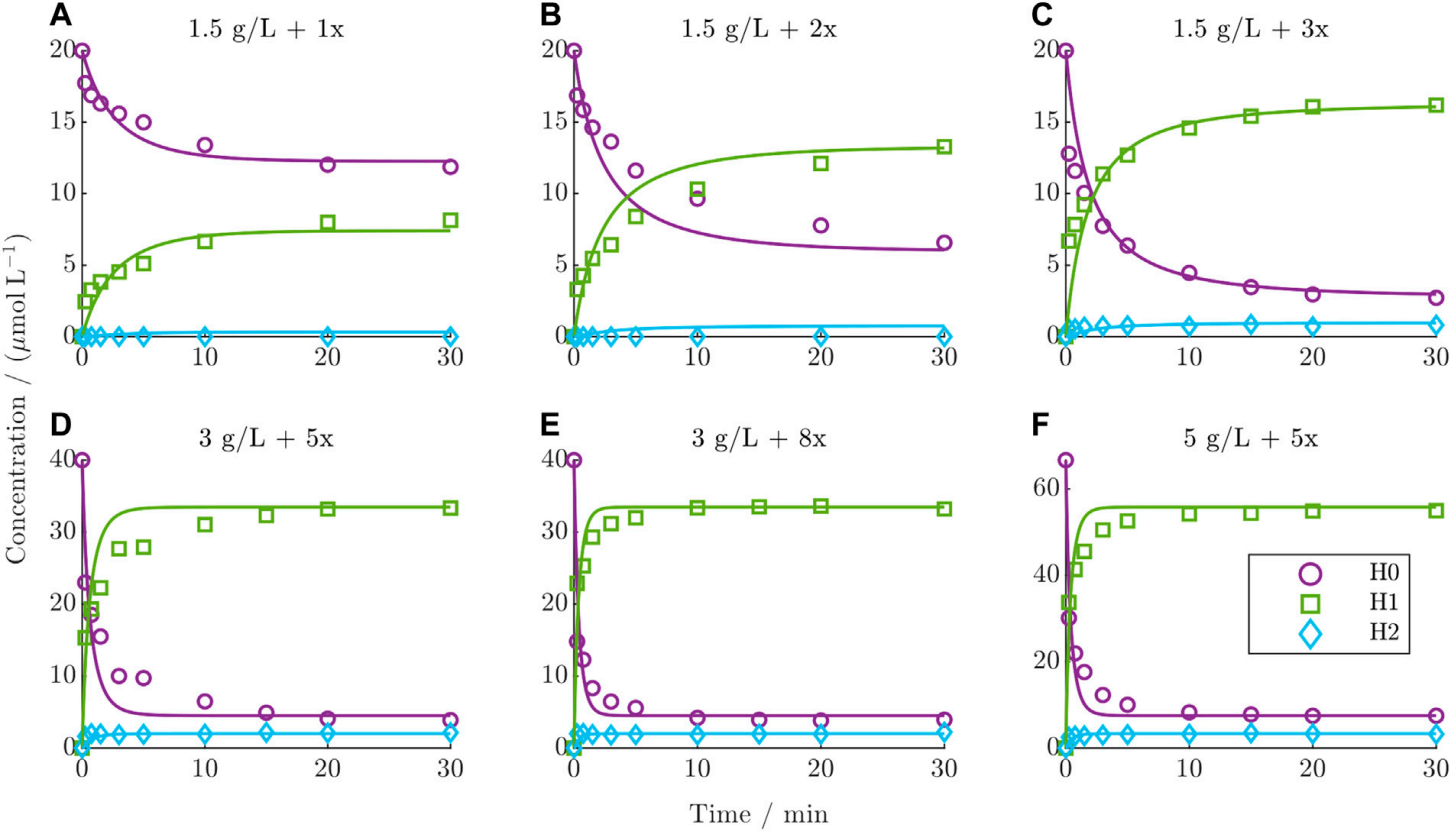
Comparison of DAR 2 model predictions vs experimental data for the six training runs using ADC1 + Drug1. **(A–F)** The mAb concentration and drug excess is shown in each title.

The dynamic behavior of the DAR 2 kinetics under various starting conditions in comparison with the predictions from the established kinetic model is depicted for the six training runs in Figure 4 (test runs are given in the Supplementary Figure S3). The experimental kinetics show a decrease in the concentration of H0 over time concurrent with the formation of H1 and H2. This trend intensifies with increasing drug excess and mAb concentration. The species H2 generally exhibits very low concentrations. Notably, the distribution of the final species depends on the drug excess. Below a drug excess of 3x, the final concentration of H0 diminishes, while H1 increases proportionally with increasing drug excess, as can be observed for the kinetics in the top row (Figures 4A–C). For a drug excess of 3x or higher (Figures 4C–F), the final composition remains consistent, which is also characterized by reaching a constant DAR plateau (see Supplementary Figure S4). This final DLD contains mainly 84% H1 on average and lower quantities of H0 and H2 at 11% and 5%, respectively. In general, the model accurately predicts the course of the reacting species, exhibiting a minor deviation only during the initial dynamic stage of the reaction. In contrast, the model precisely captures the steady state of each reaction. Overall, the averaged *R* ^2^ values over both the training and test set for H0, H1, and H2 were 95.6%, 98.4%, and 71.1%, respectively.

#### 3.3.2 Influence of different payloads

**FIGURE 5:**
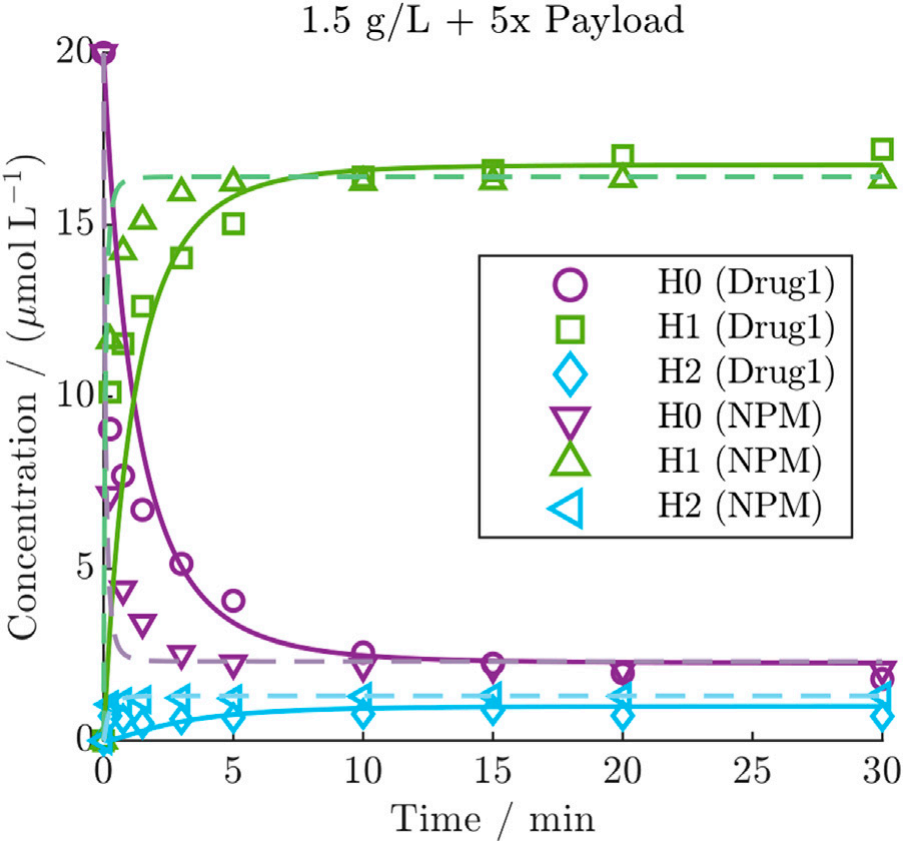
Comparison of DAR 2 kinetic with Drug1 or NPM under the same initial condition including the model predictions of the calibrated kinetic models as lines (solid line: Drug1, dashed line: NPM).

A model re-calibration on the NPM dataset yielded comparable model accuracy with averaged *R* ^2^ values of 93.1%, 94.4% and 86.1% for H0, H1, and H2, respectively. Despite the higher depletion rate of NPM, the kinetics revealed a faster conjugation compared to Drug1 under identical initial conditions, as demonstrated for one condition in Figure 5 (all NPM kinetics are provided in Supplementary Figure S5). This comparison shows that NPM reaches the steady-state more rapidly compared to the other payload, while the distribution of the final species is comparable. The observation was quantified through a comparison of the estimated conjugation rate of the two payloads with $k_{1,Drug1}=0.251\;L\;{\left(mmol\;s\right)}^{-1}$ and $k_{1,NPM}=4.840\;L\;{\left(mmol\;s\right)}^{-1}$ indicating a substantial difference in the conjugation rates.

### 3.4 Modeling interchain-cysteine conjugation kinetics

#### 3.4.1 DAR 8 batch and fed-batch conjugation

**FIGURE 6:**
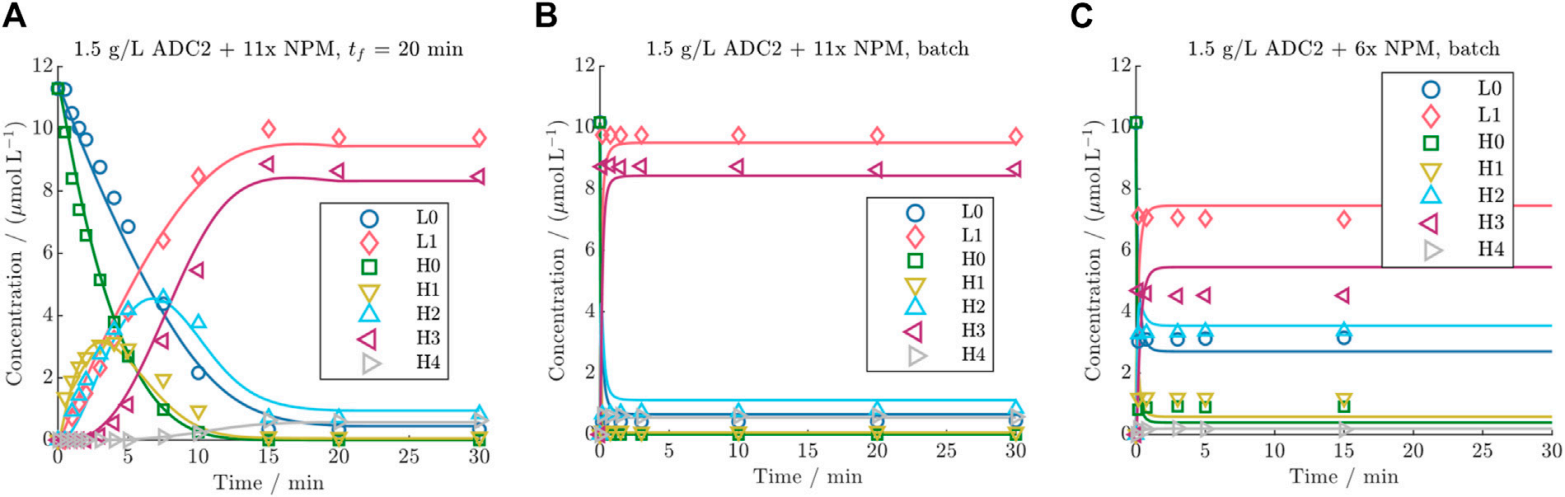
Comparison of DAR 8 model predictions vs experimental data for ADC2 at a mAb concentration of 1.5 g/L for **(A)** a fed-batch run with 20 min drug feeding time and 11x NPM, and two batch runs with **(B)** 11x NPM and **(C)** 6x NPM.

The ability of the established model to predict both DAR 8 fed-batch and batch conjugation reactions for the identical ADC was investigated in more detail. The results of three representative kinetics are presented in Figure 6 (all kinetics are given in the Supplementary Figures S6 and S7). Compared to experimental batch runs, the data from the fed-batch demonstrates that this drug addition mode results in remarkably slower reaction rates for the individual species within the initial time period of 15 min. This helps resolve the trajectories of each of the reacting species. In contrast, the samples from the batch kinetic exhibit complete conjugation already for the first time point. Furthermore, the comparison of the final steady states of all runs with a drug excess greater than 8x, suggests that a constant final species distribution is reached, as also characterized by reaching a DAR plateau (shown in Supplementary Figure S8). This distribution is characterized by L1 and H3 as the main species, with an average of 96% L1 (from total light chain) and 84% H3 (from total heavy chain). The minor species were L0, H0, H1, H2 and H4 with an average of 4% L0 and 0.01% H0, 0.5% H1, 9% H2 and 6% H4. The utilization of lower drug excesses, such as 6x, does not lead to full conjugation as can be seen in Figure 6C. Generally, the kinetic model is able to predict the kinetics of L0, L1, H0, and H3 with high run-averaged *R* ^2^ values of 0.97, 0.96, 0.98, and 0.93, respectively. However, it exhibits slightly lower run-averaged *R* ^2^ values of 0.86, 0.76, and 0.86 for predicting the kinetics of H1, H2, and H4, respectively, across all runs. This indicates a minor discrepancy between model predictions and experimental data for the species H1, H2, and H4. The predictions for all runs are provided in the Supplementary Figures S5 and S6. The estimated kinetic rates for the DAR 8 conjugation of NPM range from approx. 1.2 to 5.1 $L\;{\left(\text{mmol}\;s\right)}^{-1}$ (cf. Table 2). This demonstrates that these rates are within the same range as observed for the conjugation of the same payload in the case of DAR 2 (cf. Section 3.3.1).

#### 3.4.2 In-silico screening for influence of initial mAb and payload concentrations

**FIGURE 7:**
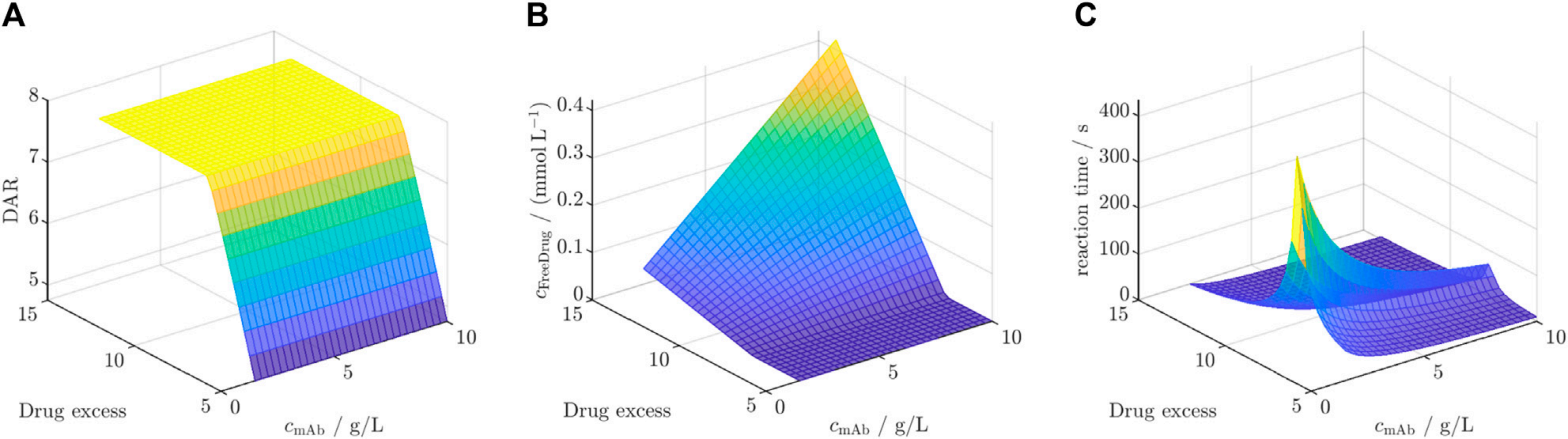
Results of the *in silico* screening for **(A)** DAR, **(B)** free unconjugated payload and **(C)** reaction time for varying initial mAb concentration $c_{mAb}$ and drug excess.

The selected DAR 8 model was first calibrated to the dataset for ADC3 + Drug2 resulting in similar kinetic rates and model fit with an *R* ^2^ of 0.99 averaged for all species (cf. Supplementary Figure S9). Afterwards, this model was employed for the *in silico* screening for DAR, free unconjugated payload, and reaction time. The results of this screening are depicted in Figure 7 for the three outputs. Figure 7A illustrates a rapid increase in DAR with escalating drug excess, independent of the mAb concentration, reaching a saturated DAR plateau at approximately 7.7x drug excess. The concentration of unconjugated payload (Figure 7B) remains at zero during the concentration range at which the DAR was found to increase, and exhibits a linear rise dependent on both variables as soon as the DAR saturation is reached. The required reaction time to achieve the final DAR (Figure 7C) begins with short reaction time of approximately 50 s, increasing with higher drug excess and decreasing mAb concentration to a maximum reaction time of 350 s. Subsequently, it decreases depending on both variables to reaction times at around 50 s.

## 4 Discussion

### 4.1 Kinetic model development

#### 4.1.1 Importance of payload depletion rate

Additional measurements to determine the payload depletion rates were necessary due to relatively high parameter uncertainties and strong correlation coefficients with the kinetic rates in models with the depletion rate as parameter to be estimated. The difference in the decrease of the UV/Vis signal for the three payloads, as observed in Figure 2, suggests that Drug1 and Drug2 are stable in conjugation buffer over the time studied here, whereas NPM becomes rapidly unavailable for participation in the reaction. Conjugations using pre-mixed NPM or Drug1 in conjugation buffer proved that NPM becomes largely unreactive within typical reaction time (1 h) as opposed to Drug1. This observation is in agreement with Andris et al. (2019), who postulate that the depletion of NPM is due to unspecific adsorption to the vessels walls or chemical inactivation. In other studies, it has been similarly proven that polycyclic aromatic hydrocarbons, such as pyrene, show high affinity to plastic in water due to chemisorption and hydrophobic interactions (Fung et al., 2023). NPM may also slowly precipitate due to its low water solubility. The involvement of multiple possible phenomena may result in the larger error bars as observed for the duplicates. For Drug1, a minor inactivation was apparent from a slight decrease of the UV/Vis signal and since the model performance was improved by the addition of a slow depletion rate. In summary, this demonstrates the need for investigating the payload behavior and stability as well as highlights the difference in depletion between the two real drugs, Drug1 and Drug2, and NPM.

#### 4.1.2 Model complexity evaluation for DAR 2 and DAR 8

For both conjugation model groups, DAR 2 and DAR 8, the parameter that exclusively accounts for the last conjugation step, had almost zero effect on the model output as shown by low parameter importance values (cf. Figure 3). This can be attributed to the consecutive reaction pathway coupled with the fact that the highest conjugated species are a low-concentrated product formed as the last species during the reaction. For instance, for DAR 2 the rate $k_{1}$ affects all reacting species, whereas the rate $k_{2}$ only partially affects the concentration of H2. In total, this results in minor contributions by the later conjugation rates to the parameter sensitivity. Therefore, the available data is not informative to estimate the rates for the over-conjugation and justifies the removal of the over-conjugation rate leading to a reduced model version with no loss in prediction error indicated by similar RMSE values for DAR 2. The decreasing trend of the influence of the later conjugation rates, as shown in Table 2, can similarly explain why the rates $k_{3}$ and $k_{4}$ in the DAR 8 model, representing the subsequent conjugations, have lower importance and larger confidence intervals. However, here the standard deviation is below 20% relative to the estimated parameter, indicating acceptable estimation quality (Sin et al., 2016). The methodology of using the parameter uncertainty and the parameter importance for the selection of proper sub-models can also be found in other works (Montes et al., 2018).

Especially when applied to model DAR 8 fed-batch conjugation, the detailed model demonstrates enhanced accuracy compared to the simple model, as depicted in Figure 3C, and supported by the reduction in RMSE values. The simple 1k model exhibits inadequacies in resolving the species trajectories during the initial stages of the reaction. In contrast, the detailed model excels in capturing the nuances of species trajectories by discerning the rates associated with each sequential conjugation step. Notably, it is observed that the conjugation to the light chain, with a rate constant ( $k_{1}$ ) of 1.408 $L\;{\left(mmol\;s\right)}^{-1}$ , is slightly slower than the first conjugation step of the heavy chain, which has a rate constant ( $k_{2}$ ) of 2.111 $L\;{\left(mmol\;s\right)}^{-1}$ . This discrepancy may be attributed to the presence of only one binding site on the light chain compared to the three available binding sites on the heavy chain. Additionally, the second conjugation step exhibits an almost twofold increase in kinetic rate compared to the first step, while the third step experiences a reduced kinetic rate. Consistent with findings in Andris et al. (2019), an ascending trend in subsequent conjugation steps is noted, associated with the increasing hydrophobicity on the mAb when the payload is bound. The final decrease in the kinetic rate in our case may be linked to the limited availability of one binding site left to react. The estimated conjugation rates ranged between ${0.3-5∙10}^{3}\;L\;{\left(mol\;s\right)}^{-1}$ which is in the expected range for rate constants of maleimides with thiols (Smith et al., 2018), and significantly faster than amine conjugation of reactive PEG with rate constants around ${1∙10}^{-1}\;L\;{\left(mol\;s\right)}^{-1}$ (Mao et al., 2022). One disadvantage of the current model is the lack of resolution for the reactivities of the different binding sites in a single molecule. This would require more sophisticated analytics, such as LC-MS (Janin-Bussat et al., 2015), in combination with advanced kinetic models which could enable the forecasting of the reactivity of specific sulfhydryl groups and how this would affect the probability of the subsequent conjugation to the other residues. For instance, Mao et al. (2022) recently presented a structure-depended reactivity model for PEGylation which enabled them to estimate the reactivity of individual amines based on molecular descriptors.

### 4.2 Insights from DAR 2 conjugationmodeling

#### 4.2.1 Modeling accuracy and analytical challenges

As shown in Figure 4, the DAR 2 model was able to model the trajectories of the heavy chains accurately, especially for H0 and H1 with *R* ^2^ values above 93%. Only the species H2 is not precisely modeled, which can be attributed to its high analytical variance owing to poor HPLC resolution of this species. It was reported that this over-conjugation is located at non-reformed disulfide bond between heavy and light chain (Cao et al., 2019). The herein established model, however, does not enable derivation of additional information about this phenomenon as this species makes up a low percentage of the total mixture, thus causing a small impact on the model predictions. Notably, the transient behavior of the distribution of the final species for varying drug excess is also captured by the model. The observation that a consistent composition is reached with around 3x drug excess, despite a stoichiometric requirement of 2x drug excess for the number of binding sites, further exemplifies the occurring payload depletion for Drug1. Additionally, the model showed a systematic discrepancy in the initial stage of the reaction. According to Andris et al. (2019), the sequential conjugation in the hinge region results in a step-wise increase in the conjugation rate due to an increase in hydrophobicity in this region. In contrast, the herein established heavy chain model lumps this phenomenon in one single rate ( $\left.k_{1}\right)$ because of an inability to account for this phenomenon due to the reducing analytical assay which removes the information about the number of payloads already bound to the intact ADC molecule. In summary, these discrepancies were considered acceptable for the purpose of modeling the DAR 2 conjugation kinetic.

#### 4.2.2 Conjugation rates difference for the utilized payloads

Calibrating the model on the two datasets using either Drug1 or NPM could show that the conjugation kinetic rate for NPM is approximately 20 times higher than for Drug1 (see Figure 5). This difference may be rather related to molecular size than hydrophobicity, as NPM has a molecular mass that is approximately five times lower than Drug1 and both drugs exhibited a similar elution time in the RP (data not shown). Pfister et al. (2015) demonstrated differences in conjugation rate depending on the size of the conjugated molecule, in the case of conjugation of PEG to lysozyme which was described using a core-shell model. In our case, the finding highlights the inequality of two payloads with regards to their conjugation kinetic rate in addition to the said difference in their depletion.

### 4.3 Insights from DAR 8 conjugationmodeling

#### 4.3.1 Modeling accuracy and exploring drug excess thresholds

The experimental data revealed that there is a drug excess threshold between 8–11x in which a consistent DLD is achieved. Using a drug excess larger than 8x did not alter the percentages in the final DLD or led to higher DAR values (cf. Supplementary Figure S8), which suggest that a critical point is reached beyond which further increases in the drug excess does not result in a higher DAR. Drug excesses below this point (e.g., 6x, see Figure 6C), lead to not reaching full conjugation which is exemplified in the lower percentages of L1 and H3 in the steady-state. Because NPM was found to simultaneously deplete in the solution, this suggests that some payloads require a drug excess higher than the stoichiometric molar quantity of binding sites to achieve full conjugation. Furthermore, the species distribution in the final steady states remained relatively constant, primarily related to the constant reduction conditions within this kinetic subset. The lower percentages of L0, H0, H1, and H2 likely originate from partially reduced species, with fewer interchain disulfides. In the literature, comparable DLDs were reported with these low levels of under-conjugated species (Källsten et al., 2018; Jones et al., 2020), and it was noted that achieving full conjugation is challenging due to the relatively low concentration of reactants (Li et al., 2020). The presence of low percentages of H4 is unique to the herein utilized IgG1 and is likely to be attributed to the said “mis-alkylation” of the payload (Paulech et al., 2013).

#### 4.3.2 Feeding mode comparison

The utilization of fed-batch runs lowered the conjugation rates enabling the elucidation of the reaction mechanism (see Figures 6A,B). Feeding times of 10 min or longer yielded satisfactory analytical resolution of the individual species. The model exhibited slightly lower accuracy for the species H0, H1, H2, and H4, which can be primarily associated to their higher analytical error due to lower concentration ranges. The batch runs emphasized that DAR 8 conjugation reactions are notably faster than DAR 2, with a time-scale of only a few minutes, compared to 5–10 min for DAR 2 reaction. Hereby it became also evident that the utilization of the complex (4k) model and the precise determination of the conjugation rates are only required when modeling fed-batch conjugation reaction. As expected, the model’s performance is primarily affected by the initial cysteine distribution when being applied for the batch reaction, as this defines the steady state of the reaction. Using the simple (1k) DAR 8 model for the prediction of the ADC3 batch kinetics yielded an identical average *R* ^2^ of 0.99 as the detailed (4k) model. This highlights the importance of accurately determining the initial cysteine distribution for the model precision, when applying the model to batch kinetics.

#### 4.3.3 Model adaptability

Moreover, it was demonstrated that the estimated kinetic rates for the same payload molecule are comparable among different ADC types in our study. This suggests the potential adaptability of the utilized modeling approach across different modalities and its potential for transfer to diverse conjugation kinetics without the necessity of prior calibration.

#### 4.3.4 In-silico screening for enhanced reaction understanding

The observed behavior in the screening for ADC3 + Drug2 reveals significant insights into the interplay of initial mAb concentration and drug excess on the studied key model outputs, as shown in Figure 7. The rapid increase in DAR until a plateau with increasing drug excess, independent of mAb concentration, aligns with the already observed saturation effect. The saturation is independent from the initial mAb concentration as the initial mAb concentration only defines the time point at which the DAR is reached. Notably, the achieved saturated DAR is achieved once the stoichiometric molar drug ratio to mAb is utilized, as Drug2 did not show to deplete and, thus, being complete available for conjugation. Similar results were reported for the DAR 2 reaction for the DAR saturation (Andris et al., 2019). The linear increase in unconjugated payload concentration beyond the DAR plateau phase suggests that, once the saturation point is reached, excess drug molecules remain unbound and contribute to the free payload concentration. Regarding the reaction time, the initial rapid increase is due to the increasing saturation DAR value below the critical drug excess of 7.7x, which requires prolonged reaction times. The maximum reaction times, aligning with DAR saturation, is achieved at low mAb concentration due to slower conjugation rates. After the DAR is saturated, both initial mAb concentration and drug excess lead to faster conjugation rates which corresponds to shorter reaction times. Overall, these findings highlight the benefit of being able to forecast the reaction behavior for various process parameters. Overall, this screening showcases the benefit of a kinetic model due to additional insights into the reaction kinetics and the reaction time-scales which is crucial for optimization of operating conditions. Especially, the knowledge of the optimal drug excess is highly useful since payloads are costly and free payload requires subsequent filtration steps in order to ensure product safety (Fernandez-Cerezo et al., 2023).

## 5 Conclusion

In this study, we developed a kinetic modeling methodology that can accurately predict both site-specific conjugations (DAR 2), and conjugations to reduced interchain disulfide bonds (DAR 8) of various payloads. A readily available RP-UHPLC method was used to acquire the kinetic data. For the kinetic model development, we covered multiple facets: First, UV/Vis measurements were performed to determine the payload stability in conjugation buffer alone. Secondly, different kinetic model candidates for the two modalities were formulated and selected. Fed-batch experiments proved to be crucial in resolving the rapid conjugation kinetics in the case of interchain disulfide conjugation. Thirdly, the kinetic model was applied to external datasets containing other payloads. A key outcome was the confirmation of major differences in both the conjugation rates and the stability of the tested pseudo and real payloads. Modeling DAR 2 and DAR 8 conjugation with the same payload demonstrated comparable rates, while for DAR 8 conjugation varying conjugation rates became apparent for the sequential conjugation steps. Following this, the calibrated DAR 8 kinetic model was used for an exemplary *in silico* screening to study the reaction behavior across a range of initial conditions. Overall, these results emphasize that this kinetic model framework is highly valuable for augmenting experimental studies, providing enhanced process understanding and optimizing the conjugation process. This approach not only complements traditional DoE methodologies but also addresses the inherent gap in DoE by offering mechanistic insights of the conjugation process, thereby accelerating process development. In future, it could be extended with other relevant influencing factors, such as pH or salt effects, or tested on other conjugation chemistries.

## Data Availability

The raw data supporting the conclusion of this article will be made available by the authors, without undue reservation.
