# Supplementary material for: Kinetic models towards an enhanced understanding of diverse ADC conjugation reactions
Source: Front Bioeng Biotechnol. 2024 Jul 11;12:1403644. doi: 10.3389/fbioe.2024.1403644 (PMC11274341; doi:10.3389/fbioe.2024.1403644)
Supplement: Supplementary file 1 [file DataSheet1.docx]

Supplementary Material

# Supplementary data

## Detailed overview of all experimental conditions

Table S1: Experimental starting conditions for the conjugation kinetics using two different conjugation types with three payloads and in different feeding modes.

| Type | ADC | Payload | $\boldsymbol{c}_{\boldsymbol{mAb}}$  $\boldsymbol{/ (mg m}\boldsymbol{L}^{\boldsymbol{-1}}\boldsymbol{)}$ | Molar drug excess | Fed-batch feeding time / s | Replicates | Subset |
| --- | --- | --- | --- | --- | --- | --- | --- |
| DAR 2 | ADC1 | Drug1 | 1.5 | 1x | - | 1 | Train |
| DAR 2 | ADC1 | Drug1 | 1.5 | 2x | - | 1 | Train |
| DAR 2 | ADC1 | Drug1 | 1.5 | 3x | - | 2 | Train |
| DAR 2 | ADC1 | Drug1 | 1.5 | 5x | - | 2 | Test |
| DAR 2 | ADC1 | Drug1 | 3 | 5x | - | 2 | Train |
| DAR 2 | ADC1 | Drug1 | 3 | 8x | - | 2 | Train |
| DAR 2 | ADC1 | Drug1 | 5 | 5x | - | 2 | Train |
| DAR 2 | ADC1 | Drug1 | 10 | 5x | - | 2 | Test |
| DAR 2 | ADC1 | NPM | 1.5 | 3x | - | 2 | External |
| DAR 2 | ADC1 | NPM | 1.5 | 5x | - | 2 | External |
| DAR 2 | ADC1 | NPM | 3 | 3x | - | 2 | External |
| DAR 2 | ADC1 | NPM | 3 | 5x | - | 2 | External |
| DAR 8 | ADC2 | NPM | 1.5 | 11x | 20 | 1 | Train |
| DAR 8 | ADC2 | NPM | 1.5 | 11x | 30 | 1 | Train |
| DAR 8 | ADC2 | NPM | 3 | 11x | 20 | 1 | Train |
| DAR 8 | ADC2 | NPM | 1.5 | 8x | 20 | 2 | Train |
| DAR 8 | ADC2 | NPM | 1.5 | 13x | 20 | 1 | Test |
| DAR 8 | ADC2 | NPM | 1.5 | 6x | 20 | 2 | Train |
| DAR 8 | ADC2 | NPM | 2.25 | 11x | 20 | 1 | Test |
| DAR 8 | ADC2 | NPM | 1.5 | 11x | 10 | 1 | Test |
| DAR 8 | ADC2 | NPM | 1.5 | 11x | - | 2 | Train |
| DAR 8 | ADC2 | NPM | 1.5 | 6x | - | 1 | Train |
| DAR 8 | ADC3 | Drug2 | 1.5 | 11x | - | 4 | External |
| DAR 8 | ADC3 | Drug2 | 1.5 | 14x | - | 2 | External |
| DAR 8 | ADC3 | Drug2 | 20 | 11x | - | 2 | External |
| DAR 8 | ADC3 | Drug2 | 20 | 14x | - | 2 | External |

## Reaction schemes

For DAR 2 and detailed reaction scheme:

Heavy chains with two cysteines: $H0_{2c}\underset{+Drug}{\underset{\to}{k_{1}}} H1_{2c}\underset{+Drug}{\underset{\to}{k_{2}}} H2_{2c}$

Heavy chain with one cysteine: $H0_{1c}\underset{+Drug}{\underset{\to}{k_{1}}} H1_{1c}$

Drug inactivation: $Drug\underset{\to}{k_{drug}}Drug_{intact}$

For DAR 8 and detailed reaction scheme:

Light chain with one cysteine: $L0_{1c}\underset{+Drug}{\underset{\to}{k_{1}}} L1_{1c}$

Heavy chain with four cysteines: $H0_{4c}\underset{+Drug}{\underset{\to}{k_{2}}} H1_{4c}\underset{+Drug}{\underset{\to}{k_{3}}} H2_{4c} \underset{+Drug}{\underset{\to}{k_{4}}} H3_{4c}\underset{+Drug}{\underset{\to}{k_{5}}} H4_{4c}$

Heavy chain with three cysteines: $H0_{3c}\underset{+Drug}{\underset{\to}{k_{2}}} H1_{3c}\underset{+Drug}{\underset{\to}{k_{3}}} H2_{3c} \underset{+Drug}{\underset{\to}{k_{4}}} H3_{3c}$

Heavy chain with two cysteines: $H0_{2c}\underset{+Drug}{\underset{\to}{k_{2}}} H1_{2c}\underset{+Drug}{\underset{\to}{k_{3}}} H2_{2c}$

Heavy chain with one cysteine: $H0_{1c}\underset{+Drug}{\underset{\to}{k_{2}}} H1_{1c}$

Drug inactivation: $Drug\underset{\to}{k_{drug}}Drug_{intact}$

## DAR 8 model ODEs

The ODEs for a fed-batch conjugation and the detailed model with 4ks are given with:

$\frac{dc_{L0_{1c}}}{dt}=-k_{1} c_{L0_{1c}} c_{\mathrm{drug}}-\frac{c_{L0_{1c}}}{V_{l}}q_{\mathrm{in}}$ (1)

$\frac{d{c_{L0}}_{0c}}{dt}=-\frac{c_{L0_{0c}}}{V_{l}}q_{\mathrm{in}}$ (2)

$\frac{dc_{H0_{4c}}}{dt}=-k_{2} c_{H0_{4c}} c_{\mathrm{drug}}-\frac{c_{H0_{4c}}}{V_{l}}q_{\mathrm{in}}$ (3)

$\frac{dc_{H0_{3c}}}{dt}=-k_{2} c_{H0_{3c}} c_{\mathrm{drug}}-\frac{c_{H0_{3c}}}{V_{l}}q_{\mathrm{in}}$ (4)

$\frac{dc_{H0_{2c}}}{dt}=-k_{2} c_{H0_{2c}} c_{\mathrm{drug}}-\frac{c_{H0_{2c}}}{V_{l}}q_{\mathrm{in}}$ (5)

$\frac{dc_{H0_{1c}}}{dt}=-k_{2} c_{H0_{1c}} c_{\mathrm{drug}}-\frac{c_{H0_{1c}}}{V_{l}}q_{\mathrm{in}}$ (6)

$\frac{dc_{H0_{0c}}}{dt}=-\frac{c_{H0_{0c}}}{V_{l}}q_{\mathrm{in}}$ (7)

$\frac{dc_{L1_{1c}}}{dt}=k_{1} c_{L0_{1c}} c_{\mathrm{drug}}-\frac{c_{L1_{1c}}}{V_{l}}q_{\mathrm{in}}$ (8)

$\frac{dc_{H1_{4c}}}{dt}=-k_{3} c_{H1_{4c}} c_{\mathrm{drug}}+k_{2} c_{H0_{4c}} c_{\mathrm{drug}}-\frac{c_{H1_{4c}}}{V_{l}}q_{\mathrm{in}}$ (9)

$\frac{dc_{H1_{3c}}}{dt}=-k_{3} c_{H1_{3c}} c_{\mathrm{drug}}+k_{2} c_{H0_{3c}} c_{\mathrm{drug}}-\frac{c_{H1_{3c}}}{V_{l}}q_{\mathrm{in}}$ (10)

$\frac{dc_{H1_{2c}}}{dt}=-k_{3} c_{H1_{2c}} c_{\mathrm{drug}}+k_{2} c_{H0_{2c}} c_{\mathrm{drug}}-\frac{c_{H1_{2c}}}{V_{l}}q_{\mathrm{in}}$ (11)

$\frac{dc_{H1_{1c}}}{dt}=k_{2} c_{H0_{1c}} c_{\mathrm{drug}}-\frac{c_{H1_{2c}}}{V_{l}}q_{\mathrm{in}}$ (12)

$\frac{dc_{H2_{4c}}}{dt}=-k_{4} c_{H2_{4c}} c_{\mathrm{drug}}+k_{3} c_{H1_{4c}} c_{\mathrm{drug}}-\frac{c_{H2_{4c}}}{V_{l}}q_{\mathrm{in}}$ (13)

$\frac{dc_{H2_{3c}}}{dt}=-k_{4} c_{H2_{3c}} c_{\mathrm{drug}}+k_{3} c_{H1_{3c}} c_{\mathrm{drug}}-\frac{c_{H2_{3c}}}{V_{l}}q_{\mathrm{in}}$ (14)

$\frac{dc_{H2_{2c}}}{dt}=k_{3} c_{H2_{1c}} c_{\mathrm{drug}}-\frac{c_{H2_{2c}}}{V_{l}}q_{\mathrm{in}}$ (15)

$\frac{dc_{H3_{4c}}}{dt}=-k_{4} c_{H3_{4c}} c_{\mathrm{drug}}+k_{4} c_{H2_{4c}} c_{\mathrm{drug}}-\frac{c_{H3_{4c}}}{V_{l}}q_{\mathrm{in}}$ (16)

$\frac{dc_{H3_{3c}}}{dt}=k_{4} c_{H3_{3c}} c_{\mathrm{drug}}-\frac{c_{H3_{3c}}}{V_{l}}q_{\mathrm{in}}$ (17)

$\frac{dc_{H4_{4c}}}{dt}=k_{4} c_{H3_{4c}} c_{\mathrm{drug}}-\frac{c_{H4_{4c}}}{V_{l}}q_{\mathrm{in}}$ (18)

$\frac{dc_{\mathrm{drug}}}{dt}=-k_{1} c_{L0_{1c}} c_{\mathrm{drug}}-k_{2} c_{H0_{4c}} c_{\mathrm{drug}}-k_{2} c_{H0_{3c}} c_{\mathrm{drug}}-k_{2} c_{H0_{2c}} c_{\mathrm{drug}}-k_{2} c_{H0_{1c}} c_{\mathrm{drug}}-k_{3} c_{H1_{4c}} c_{\mathrm{drug}}-k_{3} c_{H1_{3c}} c_{\mathrm{drug}}-k_{3} c_{H1_{2c}} c_{\mathrm{drug}}-k_{4} c_{H2_{4c}} c_{\mathrm{drug}}-k_{4} c_{H2_{3c}} c_{\mathrm{drug}}-k_{4} c_{H3_{4c}} c_{\mathrm{drug}}-k_{\mathrm{drug}}*c_{\mathrm{drug}}+\frac{c_{drug,in}}{V_{l}}q_{\mathrm{in}}$ (17)

$\frac{dV_{l}}{dt}=q_{\mathrm{in}}$ (20)

Here, $c_{H}$ and $c_{L}$represent the molar concentration of the heavy or light chain, respectively, where the number indicates the number of conjugated drugs and the index indicates the number of initial available cysteines, $c_{\mathrm{drug}}$ the molar concentration of payload, $c_{drug, in}$ the molar feed concentration of payload, $k_{j}$ the reaction rate for the jth conjugation step and $k_{\mathrm{drug}}$ the depletion rate of the specific payload, $V_{l}$ the reaction volume and $q_{in}$ the feed flow rate. For Eq. (20) the liquid volume of the sample is subtracted outside the ODE-solver as sample is only taken at discrete time points. For the simple model with 1k the kinetic rates are simplified respectively. For modeling batch reaction, the last term in Eq. (1)-(19) representing the drug feeding is removed and Eq (20) is not used. This leads to 19 ODEs for the DAR 8 batch reaction.

# Supplementary tables and figures

## Conjugation with “inactivated” NPM and Drug1


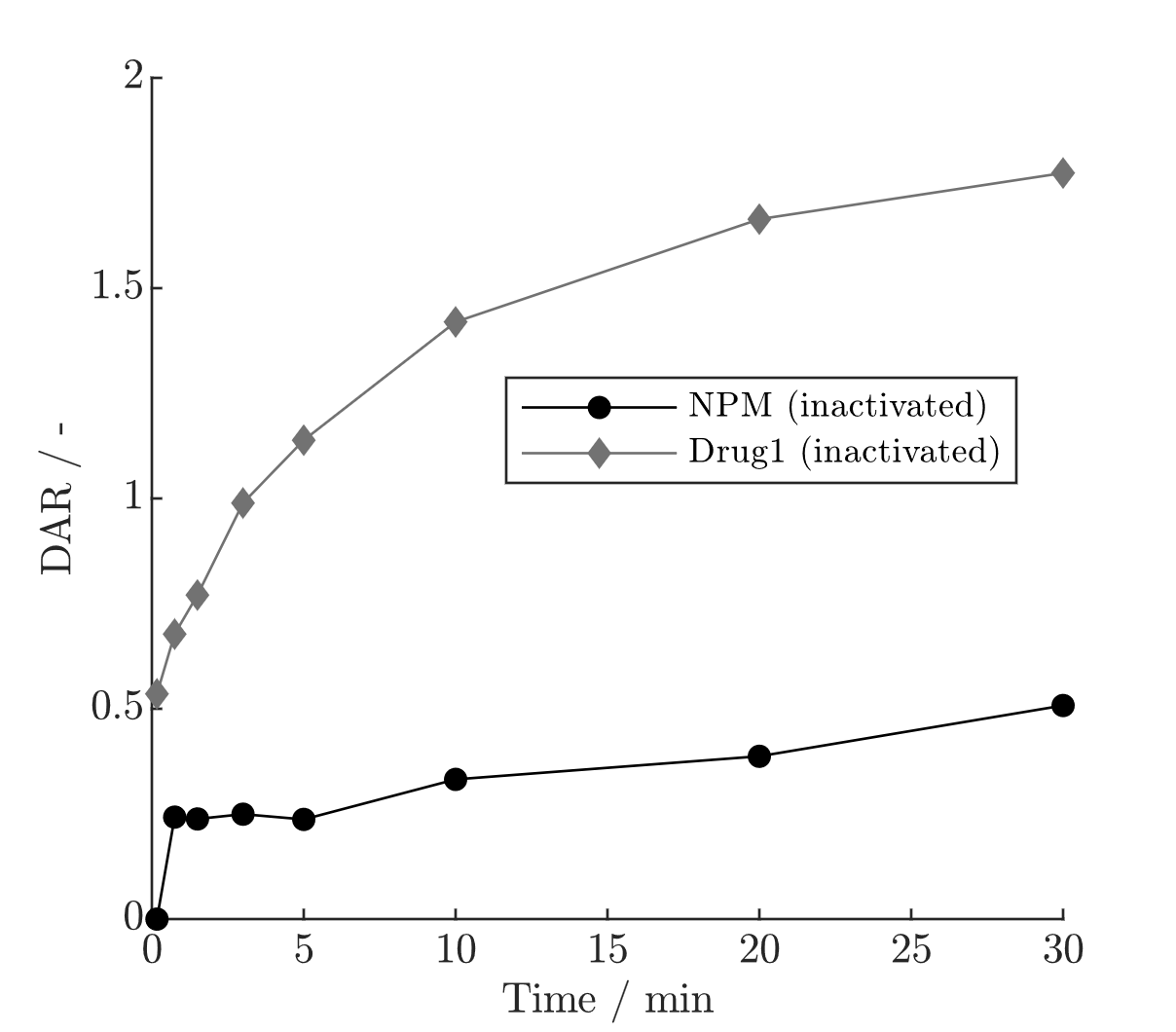


Figure S1: DAR kinetics for 1.5 g/L mAb concentration using NPM and Drug1 that were pre-mixed in conjugation buffer for one hour.

## Payload inactivation for NPM


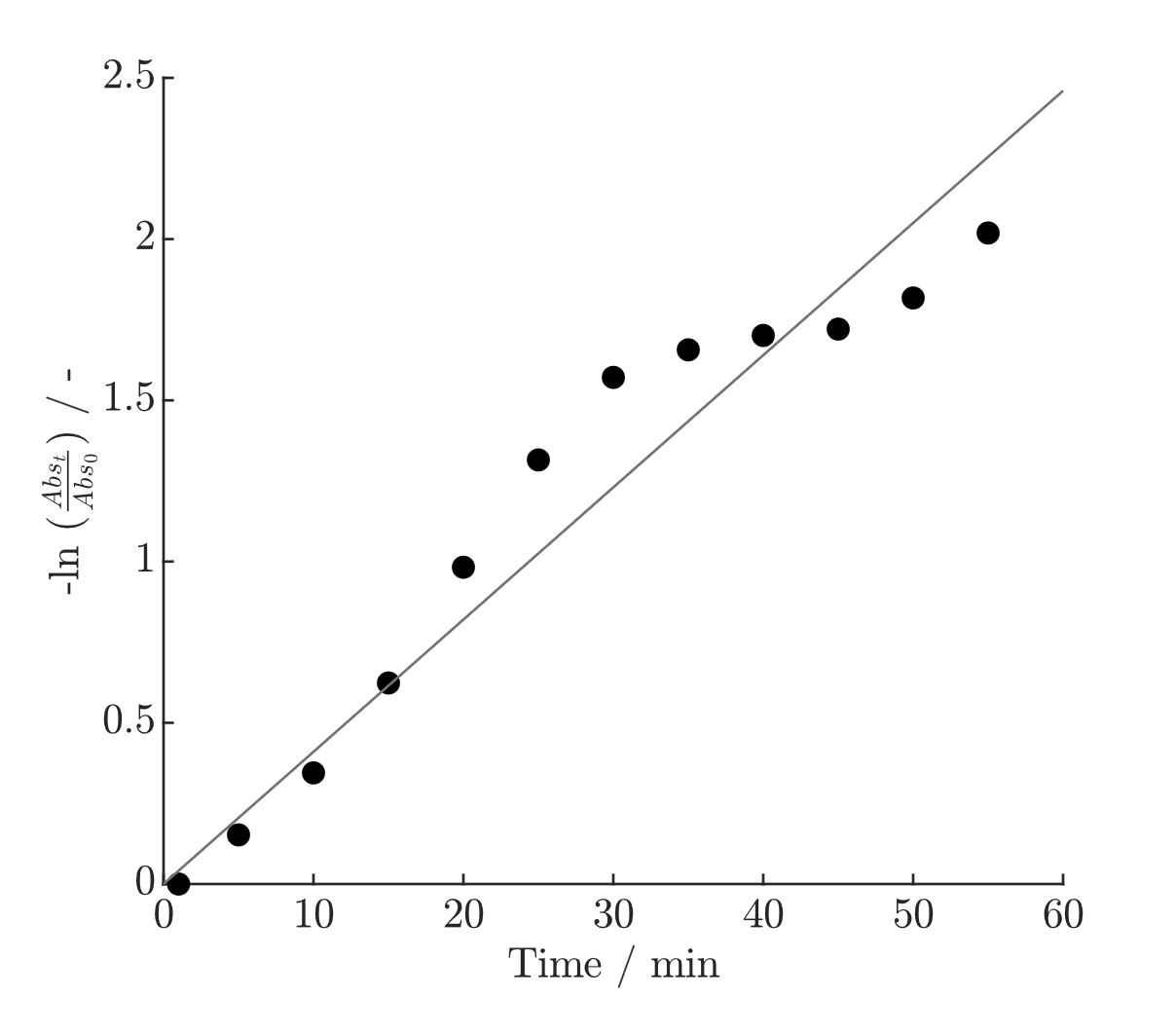


Figure S2: Linearized absorption plot of the absorbance data with a linear regression curve for NPM (R² = 0.926).

## DAR 2 model predictions for Drug1 and NPM


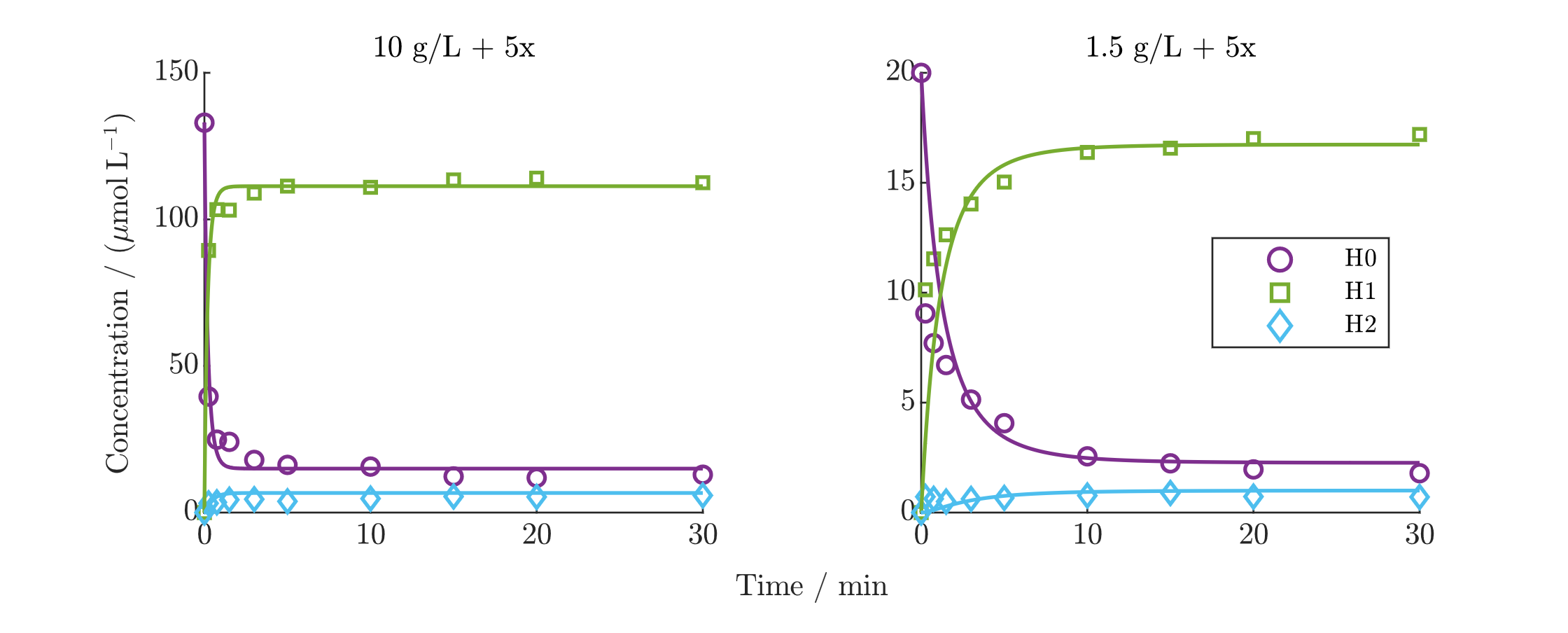


Figure S3: Comparison of DAR 2 model predictions vs. experimental data for test runs using ADC1 + Drug 1.


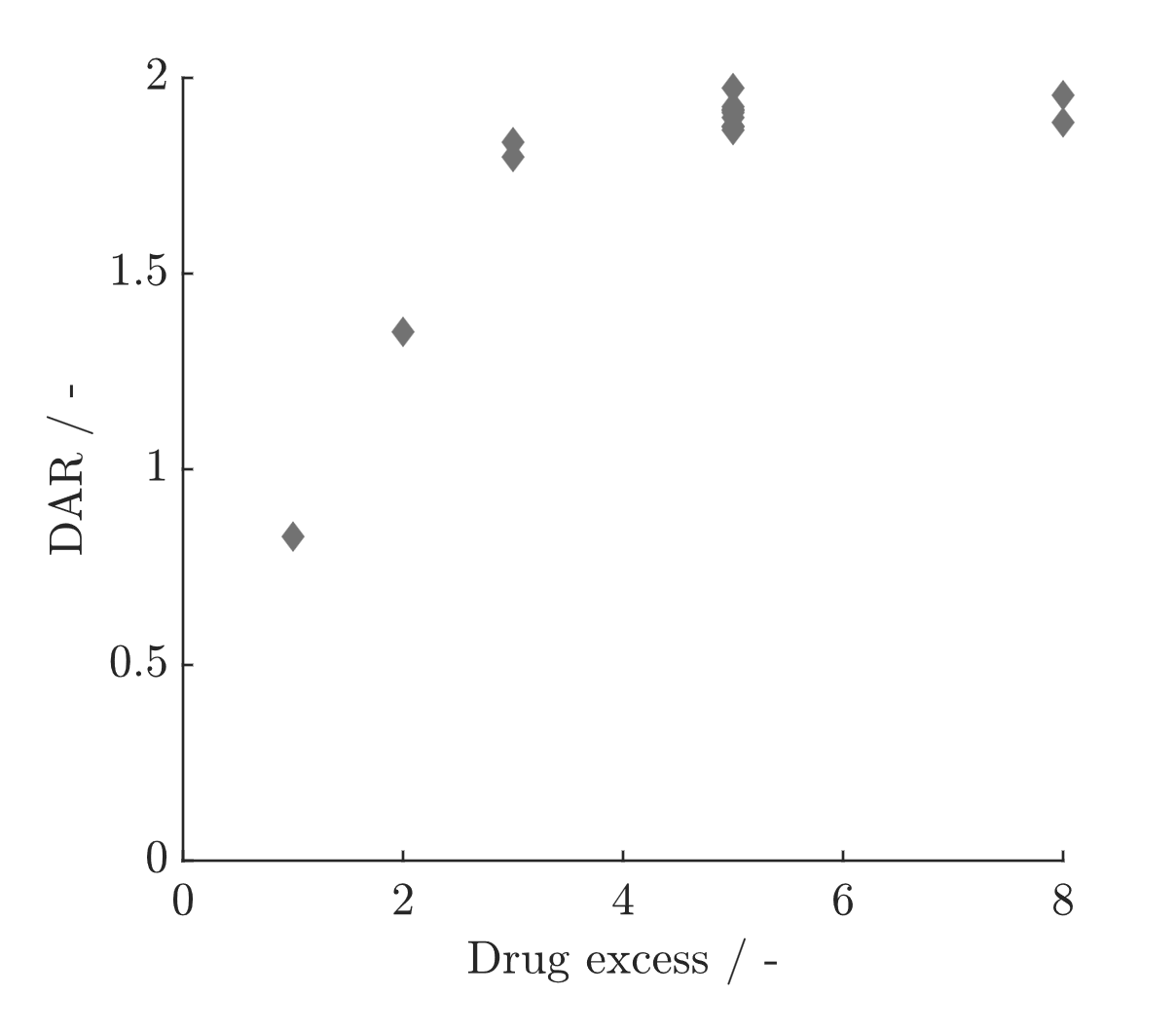


Figure S4: Final DAR values over drug excess for all ADC1 runs.


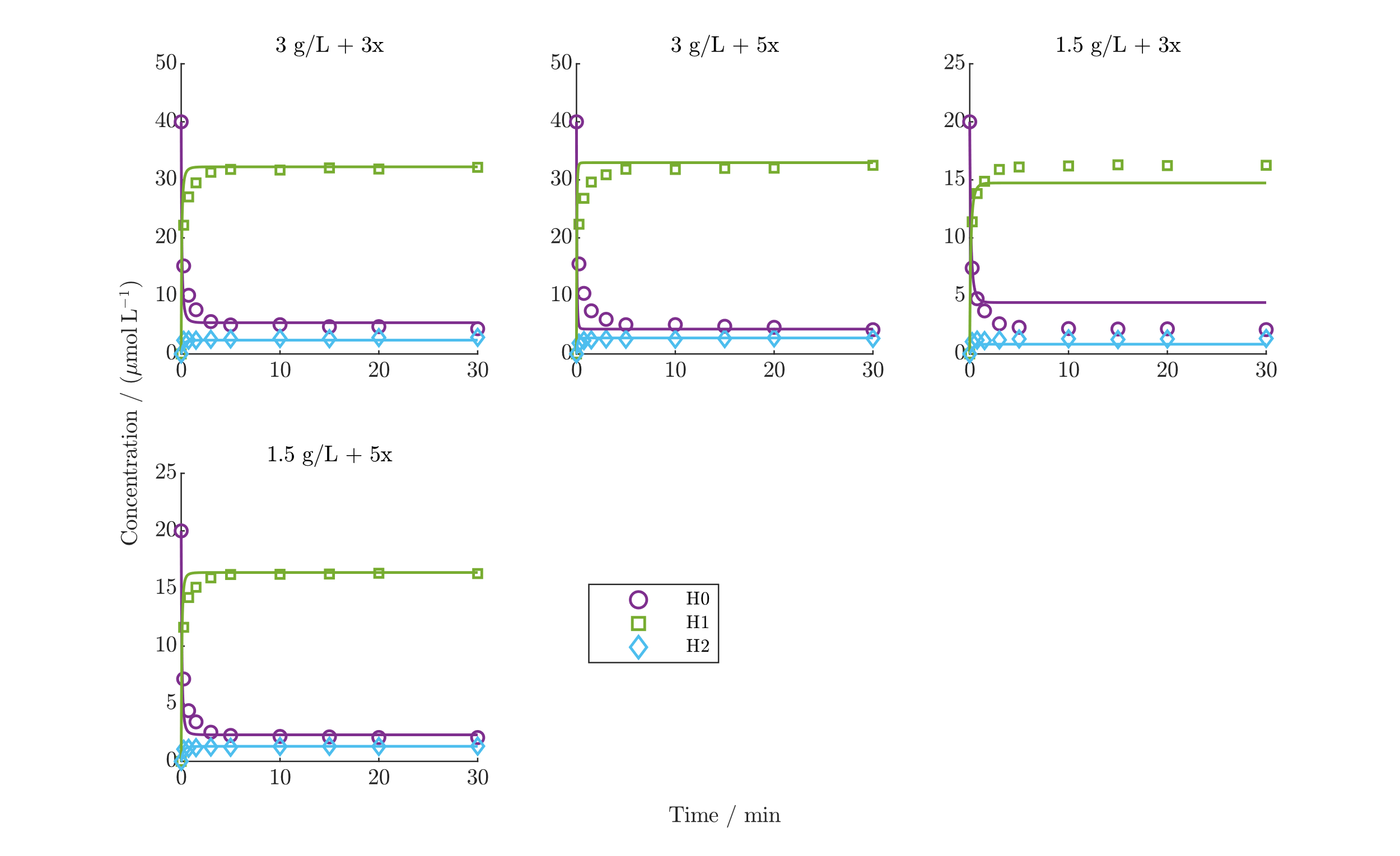


Figure S5: Comparison of DAR 2 model predictions vs. experimental data for external runs using ADC1 + NPM. Model was re-calibrated and the determined payload depletion rate for NPM was set constant.

## DAR 8 model predictions using ADC2 for both training and test set


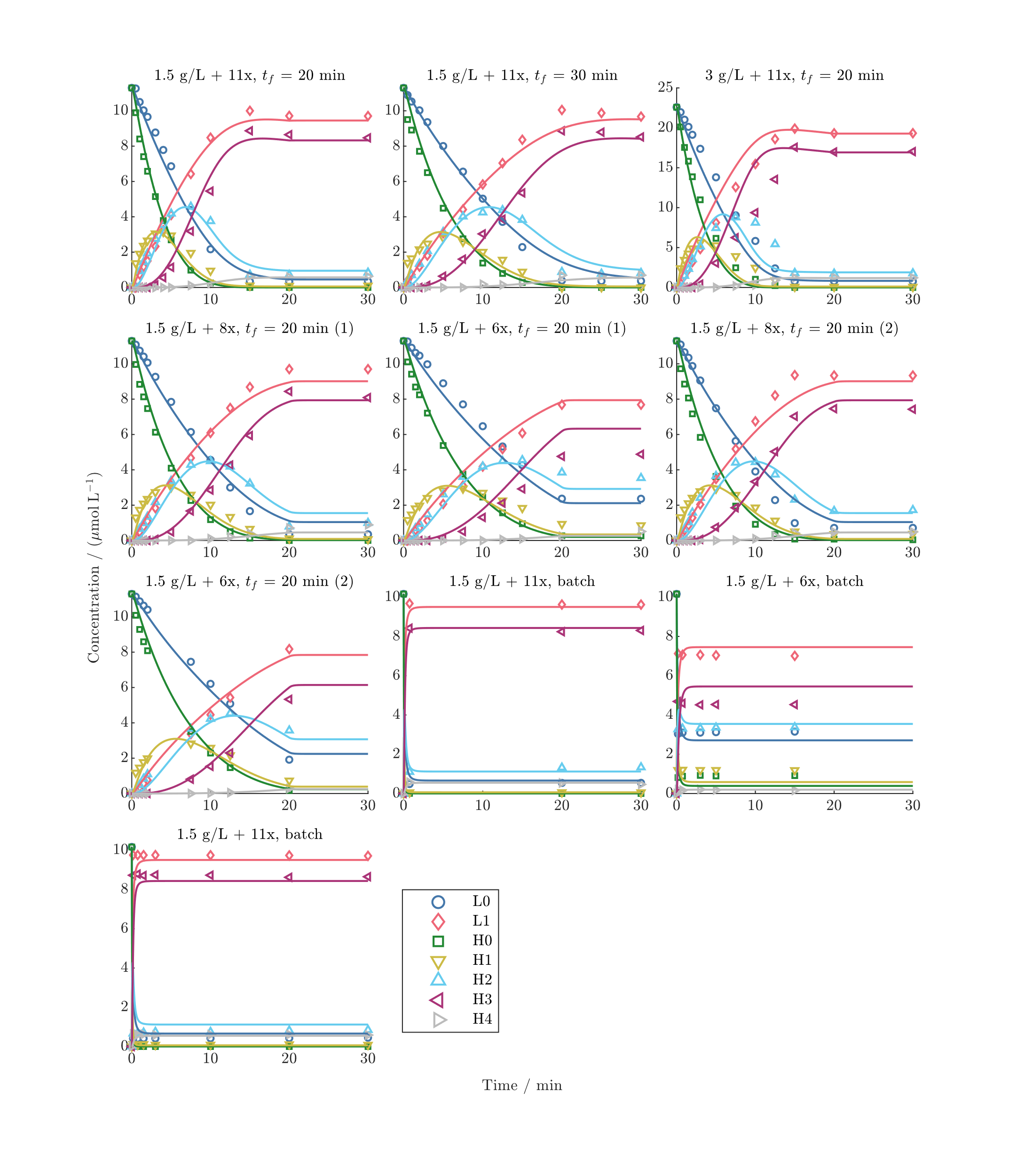


Figure S6: Comparison of DAR 8 model predictions vs. experimental data for all ten training runs of ADC2 + NPM. For duplicate runs, the replicate number is indicated in the brackets.


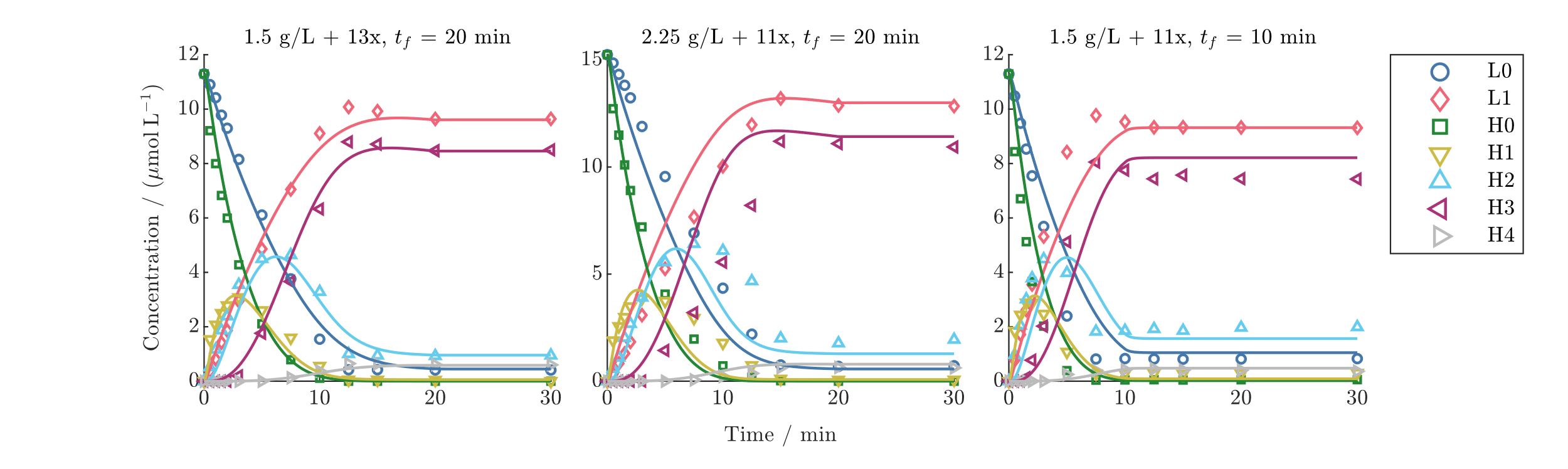


Figure S7: Comparison of DAR 8 model predictions vs. experimental data for all three test runs of ADC2 + NPM.


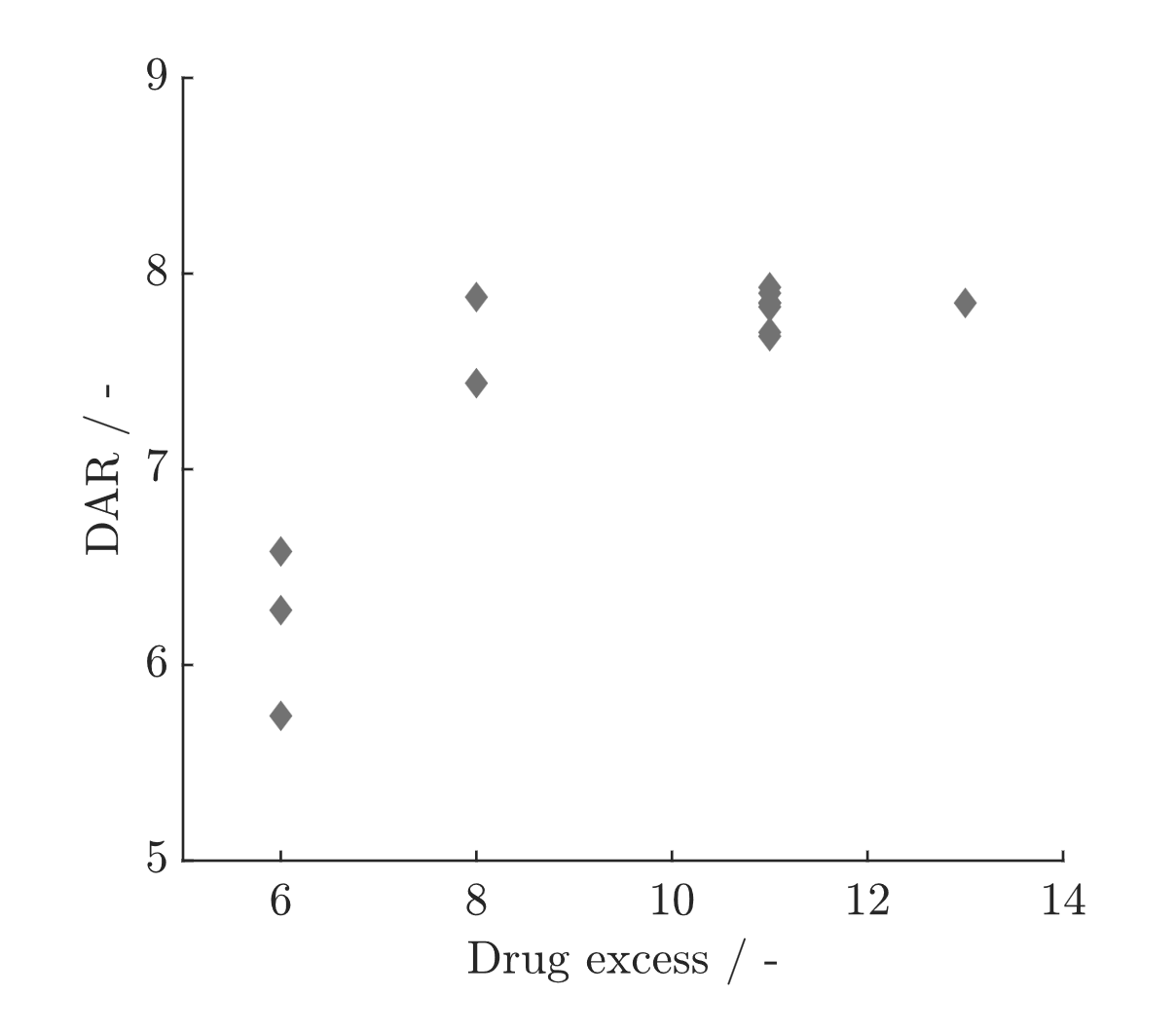


Figure S8: Final DAR values over drug excess for all ADC2 runs.

## DAR 8 model predictions using ADC3


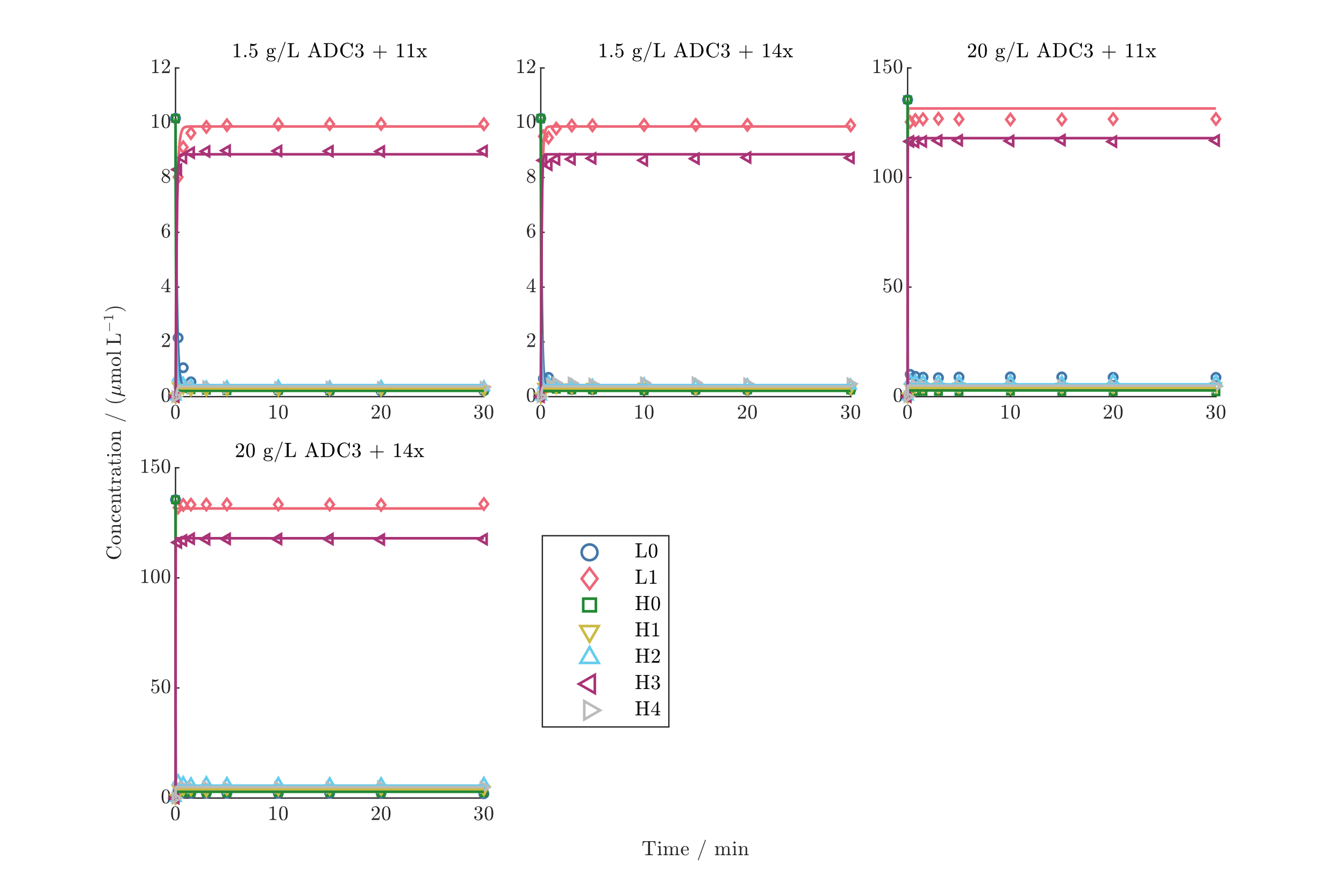


Figure S9: Comparison of DAR 8 kinetic model predictions vs. experimental data for a mixed kinetic dataset of ADC3 conjugated with Drug2. All runs were performed in duplicates, except condition 1.5 g/L ADC3 + 11x was performed in quadruplicates.
